# ZIP8 modulates ferroptosis to drive esophageal carcinoma progression

**DOI:** 10.1038/s41419-025-07692-z

**Published:** 2025-05-06

**Authors:** Zhaojie Yang, Kexin Zhao, Xiangping Li, Ruoping Yanzhang, Huijun Zhang, Yin Yu, Mingyang Yan, Shaobo Fang, Tao Li, Hao Li, Xiao Chu, Siyuan Han, Ziliang Zhang, Junyan Teng, Guoguo Jin, Zhiping Guo

**Affiliations:** 1 Henan Key Laboratory of Chronic Disease, Fuwai Central China Cardiovascular Hospital, Zhengzhou, China; 2 Laboratory of Bone Tumor, Luoyang Orthopedic Hospital of Henan Province (Orthopedic Hospital of Henan Province), Zhengzhou, China; 3 China-US (Henan) Hormel Cancer Institute, No.126, Zhengzhou, Henan China; 4 Department of Pathophysiology, School of Basic Medical Sciences, Zhengzhou University, Zhengzhou, Henan China; 5 Department of Clinical laboratory, Luoyang Orthopedic Hospital of Henan Province (Orthopedic Hospital of Henan Province), Zhengzhou, China; 6 Department of Medical Imaging, Zhengzhou University People’s Hospital& Henan Provincial People’s Hospital, Zhengzhou, China; 7 Healthy Management Center, Fuwai Central China Cardiovascular Hospital, Zhengzhou, Henan China; 8 Central China Subcenter of National Center for Cardiovascular Diseases, Henan Cardiovascular Disease Center, Fuwai Central-China Cardiovascular Hospital, Central China Fuwai Hospital of Zhengzhou University, Zhengzhou, China

**Keywords:** Bone cancer, Ion channel signalling

## Abstract

Ferroptosis, a regulated form of cell death characterized by iron-dependent phospholipid peroxidation, remains poorly understood in the context of esophageal cancer development and its regulatory mechanisms. Through comprehensive bioinformatic analyses, we identified ferroptosis-related pathways as crucial mediators in esophageal cancer progression, with ZIP8 emerging as a key regulatory element. We observed significant upregulation of ZIP8 in esophageal cancer specimens, which correlated with poor clinical outcomes. Functional studies demonstrated that ZIP8 depletion significantly attenuated cellular proliferation in vitro. Mechanistically, elevated ZIP8 expression enhanced zinc-dependent phosphorylation of CREB, leading to upregulation of the ferroptosis suppressor GPX4 and inhibition of this iron-dependent cell death modality. Significantly, we discovered that the natural compound Nobiletin targeted ZIP8, inhibiting Esophageal squamous cell carcinoma (ESCC) cell growth in vitro and in vivo. Our findings demonstrate ZIP8 as a potential therapeutic target in ESCC and suggest that promoting ferroptosis through ZIP8 inhibition may represent a novel anti-cancer strategy for ESCC therapy.

## Introduction

Esophageal cancer represents a significant global health challenge, ranking as the ninth most commonly diagnosed cancer worldwide and the sixth leading cause of cancer-related mortality. Eastern Asia displays the highest regional incidence rates [1]. Esophageal squamous cell carcinoma (ESCC) emerges as the predominant subtype, accounting for 80–90% of all cases [2]. The prognosis for ESCC remains notably poor, with 5-year survival rates critically low, falling below 20% [3]. Current treatment methods include surgery, chemotherapy, radiotherapy, and immunotherapy. However, despite these comprehensive therapeutic approaches, patient outcomes remain unsatisfactory. Consequently, identifying novel therapeutic strategies and molecular targets continues to represent a critical unmet need in esophageal cancer research.

Zinc transporters play a crucial role in maintaining zinc ion homeostasis and can be categorized into two main groups: SLC30/ZnTs and SLC39/ZIPs. These transporter proteins are the primary factors responsible for regulating the balance of zinc ions within cells [4]. In recent years, multiple studies have demonstrated the impact of the SLC39A/ZIP family on tumor development by influencing essential biological processes like cell proliferation, apoptosis, migration, and invasion [5–7]. This suggests that targeting the ZIPs family could hold promise as an effective approach for cancer treatment. The Zn transporter ZIP8 (also known as SLC39A8) is a multifunctional membrane transporter that regulates the cellular influx of divalent metals including: Zn^2+^, Fe^2+^, Co^2+^, Mn^2+^ and toxic Cd^2+^ [8–12]. ZIP8 is involved in regulation of neuroblastoma progression and metastasis [13]. Hepatic ZIP8 deficiency is associated with tumor formation [14]. ZIP8 is relatively understudied in the context of tumors, and there is a limited amount of research specifically investigating ZIP8 in ESCC.

Ferroptosis, a non-apoptotic form of cell death, has drawn significant attention from scientists in recent years as they strive to unravel its underlying mechanisms. It is believed that the excessive accumulation of lipid peroxides, generated by the lipoxygenase family, plays a crucial role in driving ferroptosis [15, 16]. This process disrupts the delicate balance maintained by glutathione and glutathione peroxidase 4 (GPX4), leading to oxidative damage and perturbation of the cellular homeostasis [17]. Compounds that inhibit the cystine glutamate transporter (system XC-) thereby reducing glutathione (GSH) levels, such as Erastin, or compounds that target GPX4 activity, such as RSL3, have demonstrated a strong ability to induce ferroptosis [15–17]. Increasing evidence underscores the pivotal role of ferroptosis as a vital tumor suppression mechanism. Notably, multiple tumor-suppressor and oncogenic signaling pathways have been demonstrated to either promote or inhibit ferroptosis, respectively [18]. To evade ferroptosis and facilitate tumor progression and metastasis, tumors have devised at least three mechanisms: they limit the synthesis and peroxidation of polyunsaturated fatty acid-phospholipids (PUFA-PLs) [19, 20], restrict the availability of labile iron [21], and upregulate cellular defense systems against ferroptosis [22–24]. Our findings illuminate the significance of ferroptosis dysregulation in esophageal cancer pathogenesis and its potential prognostic implications. By elucidating the underlying mechanisms of ferroptosis, we aim to explore the possibility of manipulating this process as a novel treatment strategy for esophageal cancer.

Currently, the exploration of anti-tumor drugs derived from plants has emerged as a crucial aspect in the field of cancer drug research [25, 26]. Nobiletin, derived from *Citrus reticulata Blanco*, is a natural extract known for its diverse pharmacological activities. These include antioxidant, anti-inflammatory, anticancer properties, as well as the regulation of cardiovascular system function and protection of the nervous system, among others [27, 28]. Nobiletin has been shown to exert inhibitory effects on the proliferation and viability of colorectal cancer cells by targeting the PI3K/AKT/mTOR signaling pathway [29]. Moreover, it stimulates apoptosis and induces cell cycle arrest in breast cancer cells by down-regulating Bcl-XL, ERK1/2, cyclinD1, AKT and mTOR, while up-regulating p21 and Bax [30]. Additionally, Nobiletin exhibits inhibitory activity against human renal cancer cells through modulation of the JAK2/STAT3 and PI3K/AKT pathways [31]. However, the molecular mechanism of Nobiletin inhibiting the growth of ESCC cells has not been reported.

Our study reveals ZIP8 as a critical oncogenic factor in ESCC. We demonstrated that ZIP8 overexpression significantly promotes ESCC cell proliferation and anchorage-independent growth in vitro. Mechanistically, elevated ZIP8 levels enhance zinc-dependent CREB phosphorylation, leading to upregulation of the ferroptosis suppressor GPX4 and subsequent ferroptosis inhibition. Notably, we identified Nobiletin, a natural compound, as an effective ZIP8 inhibitor capable of suppressing ESCC cell proliferation both in vitro and in vivo. Our comprehensive investigation unveils a novel ZIP8-mediated regulatory mechanism in ferroptosis and identifies ZIP8 as a promising therapeutic target for ESCC.

## Materials and methods

### Data process

The expression profiles associated with ESCC (GSE75241) were retrieved from the Gene Expression Omnibus (GEO) (https://www.ncbi.nlm.nih.gov/geo/). GSE75241 contained RNA expression data annotated by GPL5175, which included 15 paired ESCC samples and matched nonmalignant mucosa.

### Cell lines

The human ESCC cell lines KYSE450, KYSE150, KYSE30, KYSE510, KYSE410, KYSE140, and KYSE70 were obtained from the Chinese Academy of Sciences cell bank (Shanghai, China). The normal esophageal cell line SHEE and the embryonic kidney cell line HEK293T was obtained from Shantou University (Shantou, Guangdong, China). All cells were cultured at 37 °C in a humidified atmosphere with 5% CO_2_. RPMI-1640 medium supplemented with 10% heat-inactivated fetal bovine serum (FBS) (Biological Industries) and penicillin (100 U/ml) and streptomycin (100 ng/ml) (Meilunbio, Dalian, China) was used as the culture medium for ESCC cell lines. HEK293T was cultured in Dulbecco’s modified Eagle’s medium (DMEM).

### Reagents and antibodies

Nobiletin (CAS#478-01-3) was purchased from Shanghai Rechemscience (Shanghai, China). Z-VAD-FMK (Cat#HY-16658B), 3-MA (Cat#HY-19312), and Fer-1 (Cat#HY-100579) were obtained from MedChemExpress (Shanghai, China). Erastin (Cat#GC6630) was purchased from Glpbio Technology (Montclair, CA, United States). Puromycin dihydrochloride (Cat#IP1280) was obtained from Solarbio (Beijing, China). LipofectamineTM 2000 (Cat#11668019) was purchased from Invitrogen (Shanghai, China). Anti-ZIP8 (Cat#20459-1-AP), anti-GPX4 (Cat#14432-1-AP), and anti-Beta Actin antibodies were obtained from Proteintech Group (Chicago, IL, United States). Anti-Phospho-CREB (Ser133) (Cat#9198) and anti-CREB (Cat#9197) antibodies were purchased from Cell Signaling Technology (Danvers, MA). Anti-FTL (CAS#ab109373) and anti-FTH1 (CAS#ab183781) antibodies were acquired from Abcam (Cambridge, UK). 666-15 (GC32689) was purchased from GLPBIO (Montclair, CA, America).

### Cell counting kit-8 (CCK-8) assay

A CCK-8 kit (Meilunbio, Dalian, China) was utilized following the provided instructions to assess cell viability. Briefly, cell lines were seeded into a 96-well plate at a density of 1000 cells per well and treated with different concentrations of Nobiletin. The plate was then incubated for 24 to 96 h at 37 °C. Afterward, the culture medium was replaced with CCK-8 working solution containing 10% CCK-8 reagent. The cells were further cultured at 37 °C for 1 h. Finally, the absorbance at 450 nm in each well was measured using a microplate reader. Three replicates were performed for each cell line at each time point and growth curves were plotted.

### Colony formation assay

Cell lines were seeded into a 6-well plate at a density of 400 cells per well, followed by treatment with different concentrations of Nobiletin. The plates were then incubated at 37 °C in a 5% CO_2_ environment. Once visible colonies became apparent in the culture dish, cultivation was stopped. The culture medium was discarded, and the cells were gently washed 2–3 times with PBS. Subsequently, 1 mL of methanol was added to each well for fixation, and the fixing solution was discarded after 15 min. To stain the colonies, 1 mL of 0.1% crystal violet or Giemsa staining solution was added to each well, and the plates were stained for 10–30 min. After staining, the staining solution was slowly rinsed off with running water, and the plates were air-dried. Representative images of the stained colonies were captured and the number of colonies were counted manually. The assay was performed in triplicate for each cell line.

### Anchorage independent cell growth assay

ESCC cell lines KYSE30, KYSE450, and KYSE510 were cultured in appropriate growth medium supplemented with 10% FBS and 1% penicillin-streptomycin. Cells were maintained in a humidified incubator at 37 °C with 5% CO_2_. A solution of 0.6% agarose was prepared by dissolving agarose powder in sterile distilled water or cell culture medium. The agarose solution was autoclaved to sterilize and then cooled to ~40–45 °C. ESCC cells were detached using trypsin-EDTA solution and counted using a hemocytometer. An appropriate number of cells were suspended in complete growth medium. The cell suspension was added onto the solidified agarose-coated plates. Plates were gently swirled to ensure uniform distribution of cells and then placed back into the incubator. After 1–3 weeks, count the colonies using ImageJ Pro Plus software.

### Immunohistochemistry analysis

The ESCC tissue array, obtained from Outdo Biotech (Shanghai, China), was subjected to immunohistochemistry (IHC) staining. The tissue array was initially dewaxed in xylene and then rehydrated using a series of alcohol concentrations (100%, 95%, 75%, and 50%). Following three washes with TBST, an antigen retrieval step was performed using 10 mM citrate buffer (pH 6.0) to expose the epitopes. To block endogenous peroxidase activity, the tissues were treated with 3% hydrogen peroxide for 5 min at room temperature. Subsequently, the tissues were incubated overnight at 4 °C with a primary antibody against ZIP8 (1:100 dilution). Afterward, the tissues were incubated with an HRP-IgG secondary antibody at 37 °C for 15 min. Diaminobenzidine staining was then performed for 2 min, followed by counterstaining with hematoxylin. Representative fields on each slide were captured using an inverted microscope, and the obtained images were quantified using Image-Pro Plus software.

### Cell sample preparation and proteomics analysis

KYSE450 cells (1 × 10^6^) were treated with 40 µM Nobiletin for 24 h, after which the cells were collected for protein extraction. Digestion of protein (200 μg for each sample) was performed according to the FASP procedure [32]. Peptides were labeled with TMT reagents according to the manufacturer’s instructions (Thermo Fisher Scientific). TMT-labeled peptides mixture was fractionated using a Waters XBridge BEH130 column (C18, 3.5 μm, 2.1 × 150 mm) on a Agilent 1290 HPLC operating at 0.3 mL/min. A total of 30 fractions were collected for each peptides mixture, and then concatenated to 15 (pooling equal interval RPLC fractions). The fractions were dried for nano LC-MS/MS analysis. LC-MS analysis was performed on a Q Exactive mass spectrometer that was coupled to Easy nLC (Thermo Fisher Scientific). The resulting LC-MS/MS raw files were imported into Proteome Discoverer 2.4 software (version 1.6.0.16) for data interpretation and protein identification against the database.

### Computer docking model

PubChem served as the basis for the three-dimensional (3D) Nobiletin structure, and ZIP8 structure derived from PDB database. AutoDockTools-1.5.7 software was used to simulate docking, and select the genetic algorithm parameters. Pymol was used to prepare and analysis molecular docking.

### Drug affinity responsive target stability (DARTS) assay

The DARTS method was performed following a procedure previously described [23]. Cells were collected and lysed with lysis buffer containing protease inhibitor and phosphatase inhibitor. The lysis buffer was then supplemented with TNC buffer. The lysates were aliquoted into 1.5 ml tubes and then incubated with different concentration of tangeretin or DMSO for 1 h at room temperature. For exactly 30 min, the lysates were digested with 1 μg of pronase for every 300 μg of lysate. Then, protein loading buffer was immediately added, the lysates were then heated to stop proteolysis. Western blotting was performed as previously described.

### Cellular thermal shift assay (CETSA)

KYSE450 cells were treated with Nobiletin for 30 min. The cells were collected into PCR tubes (100 μL) and incubated at a series of temperatures from 37 °C to 64 °C, with a gradient of 3 °C for 3 min. After being frozen by using liquid nitrogen and thawed on ice for twice, the supernatant was collected for the subsequent western blotting.

### Solvent-induced protein precipitation (SIP) assay

For the SIP assay, ESCC cells were lysed and incubated with DMSO or Nobiletin. The five replications were treated with an organic solvent mixture comprising acetone: ethanol: acetic acid at a ratio of 50: 50: 0.1 to reach a final solvent concentration of 16%. Subsequently, the mixtures were equilibrated at 800 rpm for 20 min at 37 °C. The supernatants were used for Western-blotting analysis.

### Western-blotting analysis

Whole cell lysates were prepared by treating cells with RIPA buffer supplemented with a protease inhibitor (MCE). The cellular extracts were then centrifuged at 13,000 *g* for 10 min at 4 °C. The protein concentration in the supernatant was determined using a BCA protein assay kit (PIERCE). A total of 20 mg of cell lysates were boiled for 10 min and separated by SDS-PAGE. The proteins were then transferred onto a PVDF membrane. Standard western blotting procedures were carried out using primary antibodies against ZIP8 (Santa Cruz), CREB (proteintech), pCREB (proteintech), GPX4 (proteintech), FTL (proteintech), and FTH1 (proteintech). The membrane was subsequently incubated with an HRP-conjugated secondary antibody (Santa Cruz) for 1 h. After washing with PBST, the protein bands were detected by using the Western Bright ECL reagent (Advansta) and visualized with a ChemiDoc Touch Imaging System (Bio-Rad).

### Plasmid construction, transfection, and lenti-virus transduction

KYSE30, KYSE450, and KYSE510 cell lines were transfected with short hairpin RNA (shRNA) targeting ZIP8. The shZIP8 sequences were cloned into the lentiviral expression vector plko.1. The specific shZIP8 sequences used in this study were as follows: shZIP3#: 5’-CCG GCC TTG CTA TTC AAC TTC CTT TCT CGA GAA AGG AAG TTG AAT AGC AAG GTT TTT G-3’ shZIP4#: 5’-CCG GGC TGC ACT TCA ACC AGT GTT TCT CGA GAA ACA CTG GTT GAA GTG CAG CTT TTT G-3’. The transfection process was performed following a previously established protocol (reference to previous study) [33]. The efficiency of transduction was assessed by Western blot analysis, confirming the knockdown of ZIP8 protein expression. Cell growth and colony formation ability were compared between the shZIP8-transfected cells and mock-transfected cells to evaluate the impact of ZIP8 knockdown on these cellular processes. ZIP8 overexpressing vector was purchased from Youbao Bio.

### MDA measurement

Intracellular concentrations of malondialdehyde (MDA) were determined using a Cellular Lipid Peroxidation MDA Assay Kit (Cat#A003-1, Nanjing Jiancheng Bioengineering Institute, Jiangsu, China). Following the indicated treatments, ESCC cells were harvested. The measurement of MDA was conducted in accordance with the manufacturer’s instructions provided with the assay kit.

### Erastin sensitivity assay

Seed cells in plates and reach optimal growth conditions. Prepare a stock solution of Erastin in a suitable solvent according to manufacturer instructions. Add the Erastin solution to the culture medium at different concentrations (0 µM, 0.01 µM, 0.1 µM, 1 µM, 10 µM) to create a dose-response curve. Incubate the cells with Erastin for a specified time period 24 h. The viability of the cells was evaluated using CCK-8.

### FerroOrange assay

Prepare the FerroOrange staining solution by following the manufacturer’s instructions. Remove the culture medium from the treated cells and wash them gently with warm phosphate-buffered saline (PBS). Add the prepared FerroOrange staining solution to each well or plate containing the cells. Incubate the cells with FerroOrange staining solution for 30 min at room temperature, protected from light. Wash the cells again with warm PBS to remove excess stain. Visualize the stained cells using fluorescence microscopy equipped with appropriate filters for the excitation and emission wavelengths of FerroOrange. Measure immunofluorescence intensity using Image Pro Plus software, with each experiment completed independently three times.

### C11-BODIPY assay

Cells were incubated for 48 h under standard culture conditions. Prepare the C11-BODIPY staining solution by following the manufacturer’s instructions. Remove the culture medium from the treated cells and wash them gently with warm PBS. Add the prepared C11-BODIPY staining solution to each plate containing the cells. Incubate the cells with C11-BODIPY staining solution for 30 min at room temperature, protected from light. Wash the cells again with warm PBS to remove excess stain. Visualize the stained cells using fluorescence microscopy or a fluorescence plate reader equipped with appropriate filters for the excitation and emission wavelengths of C11-BODIPY. Measure immunofluorescence intensity using Image Pro Plus software.

### Cellular thermal shift assay

KYSE450 cells were treated with Nobiletin for 30 min. The cells were collected into PCR tubes (100 μL) and incubated at a series of temperatures from 37 °C to 64 °C, with a gradient of 3 °C for 3 min. After being frozen by using liquid nitrogen and thawed on ice for twice, the supernatant was collected for the subsequent western blotting.

### Detection of lipid peroxidation by flow cytometry

To evaluate lipid peroxidation, we employed the BD FACSAria II flow cytometer in conjunction with the BODIPY™ 581/591 C11 probe, known for its selective binding to lipid peroxides. The BODIPY™ 581/591 C11 probe was sourced from Maokangbio, China, with the product code MX5211-1MG. Cells were cultured under standard conditions until they achieved 70–80% confluence. To induce lipid peroxidation, cells were treated with Nobiletin or subjected to ZIP8 knockdown for a duration of 24 h. Post-treatment, cells were harvested using trypsinization, rinsed twice with chilled PBS, and then resuspended in 500 μL of PBS. Subsequently, 5 μL of the BODIPY™ 581/591 C11 stock solution (prepared at a concentration of 2 mM in DMSO) was added to the cell suspension, which was then incubated for 30 min at 37 °C in a dark environment to allow for probe binding. The stained cells were subsequently analyzed using the BD FACSAria II with the following configuration: excitation at 488 nm laser line and emission detected through a 515–545 nm bandpass filter (FL1 and FL2 channel). Data analysis was conducted using BD FACSDiva software, where gates were applied to exclude debris and doublets, and the mean fluorescence intensity of C11-positive cells was determined. To ensure the reliability of fluorescence detection, the BD FACSAria II was calibrated daily as per the manufacturer’s guidelines.

### Prussian blue staining for iron accumulation in ESCC cells

We used the Solarbio kit (G1428) to detect iron accumulation in KYSE30 cells. According to the kit’s instructions, for KYSE30 cell samples, cells were fixed with 4% paraformaldehyde for 20 min. Before use, reagents A1 and A2 were mixed in equal amounts to prepare Perls Stain. Perls Stain was then added dropwise to the cell samples and stained at 37 °C for 30 min. The samples were thoroughly washed twice with distilled water for 2 min each time. Subsequently, the cell nuclei were counter-stained with nuclear fast red or eosin staining solution for 30 s, followed by a 5 s rinse with tap water. After staining, the samples were air-dried and observed under a microscope.

### Patient-derived xenograft (PDX) mouse model

The research protocol was granted approval by the Zhengzhou University Institutional Animal Care and Use Committee located in Zhengzhou, Henan, China. We procured six-week-old female SCID mice from Vital River Labs in Beijing, China, and housed them under a 12/12 h light/dark cycle, providing them with unrestricted access to food and water. We dissected PDX tumor tissue into 1–2 mm fragments and transplanted these into the right flanks of the mice. To reduce variability in our experiments, we selected 10 mice for each group. Once the average tumor volume reached approximately 100 mm^3^, the mice were allocated randomly into two groups, each comprising 10 mice: one group received a vehicle control (0.9% saline), the second group was treated with Nobiletin at a dose of 30 mg/kg, and the third group was treated with Nobiletin at a dose of 60 mg/kg. We monitored tumor volume and body weight twice weekly, calculating tumor volume. Upon reaching a tumor volume of 800 mm^3^, the mice were euthanized, and the tumors were harvested. A portion of the tumors was preserved in formalin for hematoxylin and eosin (HE) staining and immunohistochemistry (IHC) analysis, while the remainder was stored at −80°C for subsequent protein analysis.

### Statistical analyses

SPSS 23.0 was used for statistical analyses in this study, and quantitative data were presented as the mean ± SD. To compare significant differences, the one-way analysis of variance (ANOVA), unpaired t-test and non-parametric comparisons method was used. For each experiment, *p < 0.05, **p < 0.01, and ***p < 0.001 were chosen to indicate significance.

## Results

### Identification of ZIP8 as a significant factor in ESCC and ferroptosis

**Fig. 1:**
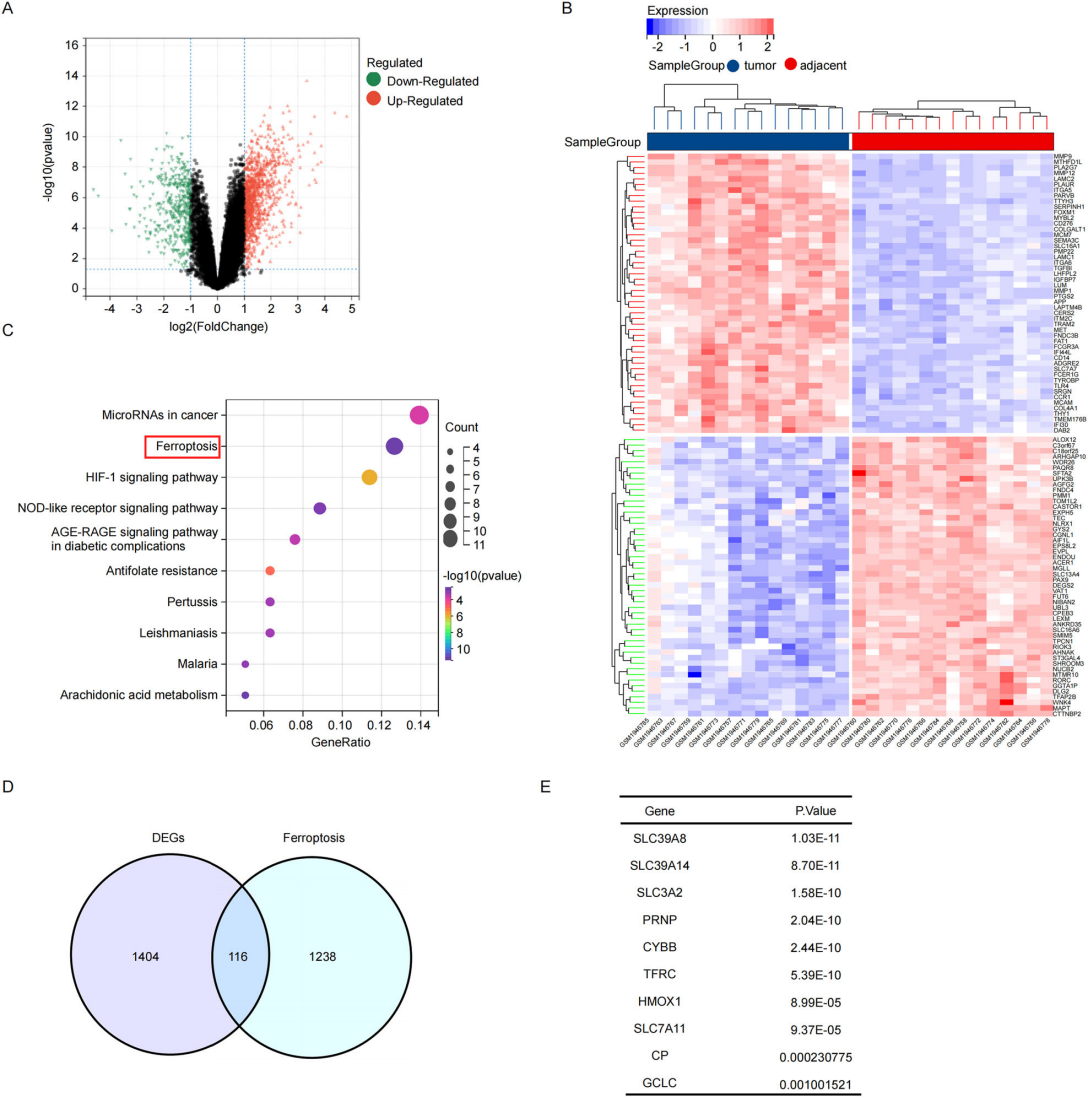
Identification of ZIP8 as a significant factor in ESCC and ferroptosis. **A** Differential gene expression analysis using GSE75241 dataset identified 1152 genes with significant differential expression under the criteria of *p* < 0.01 and logFC > 2. A Volcano plot displayed the differential expression between ESCC and normal groups derived from GSE75241. **B** A heatmap represents the top 50 differentially expressed genes (DEGs), highlighting their expression patterns across samples. **C** KEGG pathway analysis was conducted to elucidate the potential biological functions of the DEGs. **D** Venn diagram showing the intersection between genes associated with ferroptosis and the DEGs identified in our study. **E** A list of genes with their respective *p* values, indicating the statistical significance of their differential expression.

To identify key genes related to ESCC, we performed differential expression analysis on the GSE75241 dataset using stringent selection criteria (*p* < 0.01 and log2 fold change >2). This dataset comprises transcriptome profiles from 15 paired ESCC and adjacent normal tissue specimens collected from Brazilian patients. The differential expression analysis identified 1520 significantly dysregulated genes visualized in a volcano plot (Fig. 1A). Hierarchical clustering analysis of the top 50 differentially expressed genes (DEGs) is presented as a heatmap (Fig. 1B). To elucidate the functional implications of these transcriptional alterations, we conducted pathway enrichment analyses. Notably, KEGG pathway analysis revealed significant enrichment in ferroptosis-related pathways (Fig. 1C). To identify ferroptosis-associated genes specifically relevant to ESCC, we performed intersection analysis between established ferroptosis regulators and our identified DEGs, yielding 116 overlapping candidates (Fig. 1D). Among these genes, ZIP8 emerged as the most statistically significant candidate based on differential expression analysis (Fig. 1E). Our findings suggest that ferroptosis pathways potentially represent a critical mechanism in ESCC progression. Specifically, ZIP8 emerges as a potential driver gene in ferroptosis, potentially contributing significantly to ESCC pathogenesis.

### Up-regulation of ZIP8 expression correlates with poor prognosis in ESCC

**Fig. 2:**
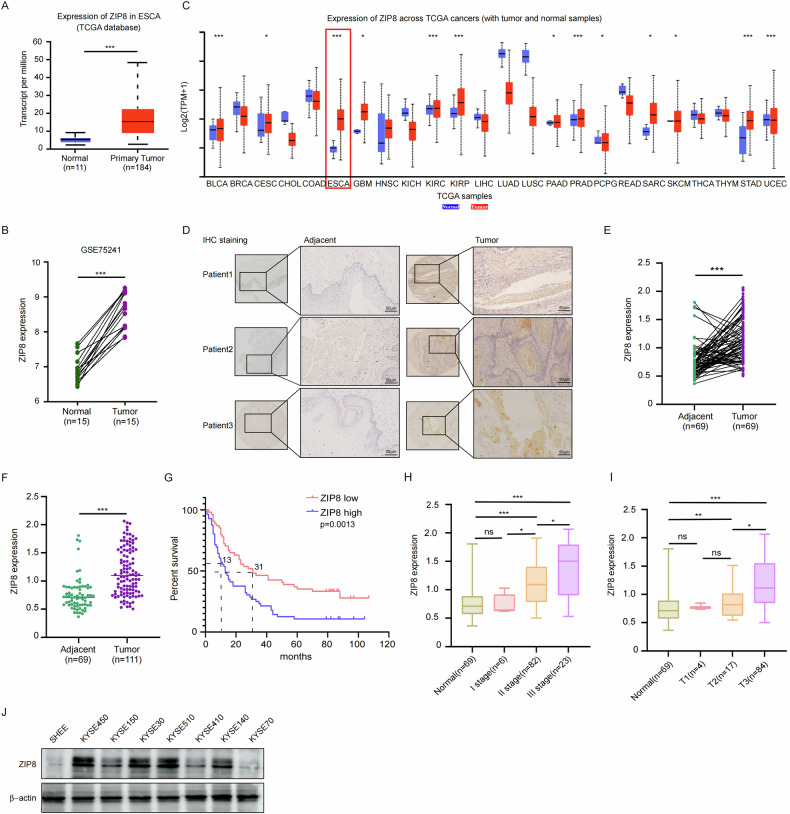
Up-regulation of ZIP8 expression correlates with poor prognosis in ESCC. **A** Analysis of ZIP8 expression in esophageal cancer using The Cancer Genome Atlas (TCGA) database. **B** GSE75241 dataset was utilized to further analyze ZIP8 expression levels. **C** ZIP8 expression profiles across various cancer types within the TCGA database. **D** Immunohistochemical (IHC) staining of ESCC tissue microarrays (20×). Staining of adjacent normal and paired ESCC tissues was performed with ZIP8 antibody. Scale bar represents 50 µm. Quantitative analysis of ZIP8 protein expression levels in paired (**E**) and unpaired (**F**) ESCC tissues from the tissue microarrays. **G** Survival analysis of cancer patients with high or low ZIP8 protein levels in tissue microarrays. Survival curves were generated using the Kaplan–Meier method. *p* value was calculated based on log-rank test. **H** Correlation between ZIP8 protein levels and clinical stage in ESCC. **I** The connection between the molecular analysis of ZIP8 protein expression and its relevance to the TNM staging system. **J** Western blot was utilized to verify the expression levels of ZIP8 in a panel of ESCC cell lines. *p* values are from Student’s unpaired (**E**–**I**) t test. Statistical significance is denoted by *(*p* < 0.05), **(*p* < 0.01), and ***(*p* < 0.001) compared to the control group (*n* = 3, independent experiments). Data are presented as mean ± SD.

To investigate the oncogenic role of ZIP8 in ESCC, we analyzed the TCGA database, which revealed significantly elevated ZIP8 expression in esophageal cancer tissues compared to adjacent normal tissues (Fig. 2A). Complementary analysis of the GEO dataset GSE75241 confirmed these findings, demonstrating high ZIP8 expression in esophageal cancer specimens (Fig. 2B). Further exploration of the TCGA database identified ZIP8 protein overexpression across multiple cancer types, including bladder cancer (BLCA), cervical cancer (CESC), esophageal cancer (ESCA), glioblastoma (GBM), kidney cancers (KIRC, KIRP), pancreatic cancer (PAAD), prostate cancer (PRAD), Pheochromocytoma and Paraganglioma (PCPG), Sarcoma (SARC), Skin Cutaneous Melanoma (SKCM), Stomach adenocarcinoma (STAD) and Uterine Corpus Endometrial Carcinoma (UCEC) (Fig. 2C). To validate these database-derived observations, we conducted immunohistochemistry (IHC) on ESCC patient tissues. The IHC analysis consistently demonstrated higher ZIP8 protein levels in tumor tissues compared to adjacent normal tissues (Fig. 2D). ZIP8 also highly expressed in paired or unpaired esophageal cancer tissues (Fig. 2E, F). Notably, high ZIP8 expression correlated with poor patient prognosis and showed significant associations with histopathological grading and clinical staging of esophageal cancer (Fig. 2G–I). Western blot analysis further substantiated these findings, revealing markedly elevated ZIP8 expression in multiple ESCC cell lines (KYSE450, KYSE30, and KYSE510) relative to the normal esophageal epithelial cell line SHEE. However, KYSE70 and KYSE410 cell lines exhibited ZIP8 expression levels similar to the SHEE baseline (Fig. 2J). Collectively, these comprehensive analyses indicate that ZIP8 is consistently overexpressed in ESCC cell lines and patient tissues, with its expression closely linked to cancer progression and patient outcomes.

### Knockdown of ZIP8 impairs ESCC cell proliferation

**Fig. 3:**
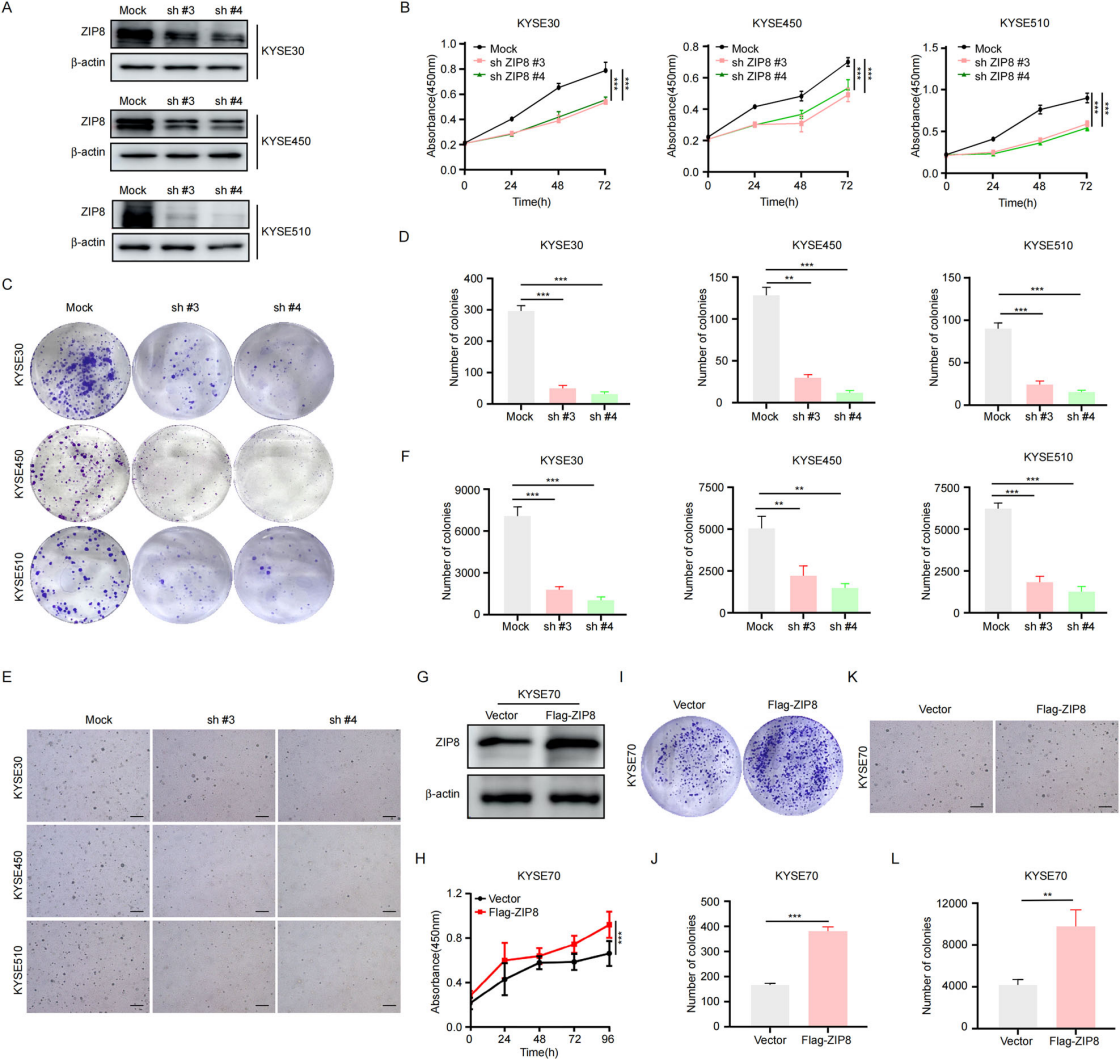
Knockdown of ZIP8 impairs ESCC cell proliferation. **A** Western blot analysis confirmed the efficiency of ZIP8 knockdown in ESCC cell lines. **B** CCK-8 assays was used to detect the proliferation of KYSE30, KYSE450, and KYSE510 cells. **C**, **D** Colony formation assays assess the ability of KYSE30, KYSE510, and KYSE450 cells to survive and form colonies following ZIP8 knockdown. **E**, **F** Anchorage-independent cell growth assays evaluate the effects of ZIP8 knockdown on cell growth (scale bar, 200 μm). **G** Western blot to check the overexpression of ZIP8 in KYSE70 cells. **H** CCK8 assays to detect the cell proliferation after overexpression ZIP8. **I–L** Colony formation assays and anchorage-independent growth assays were conducted to evaluate the effect of ZIP8 overexpression on the proliferative capacity of KYSE70 cells. Representative images are shown in the upper panel, while statistical analysis is presented in the lower panel. The Student’s unpaired t-test was utilized to evaluate statistical analysis in (**B**, **D**, **F**, **H**, **J**, **L**, with *p < 0.05, **p < 0.01, *p* < 0.001* (*n* = 3, inde*p*endent experiments). Data are presented as mean ± SD.

To elucidate the role of ZIP8 in ESCC tumor progression, we used shRNA to knockdown ZIP8 expression in esophageal cancer cell lines, including KYSE30, KYSE450, and KYSE510 cells. Western blot analysis confirmed efficient ZIP8 protein knockdown (Fig. 3A). CCK-8 assays confirmed that cell proliferation was inhibited after ZIP8 knockdown (Fig. 3B). Colony formation assays revealed a dramatic decrease in colony numbers after ZIP8 knockdown (Fig. 3C, D), while anchorage-independent growth studies further verified these findings by showing substantially impaired colony formation following shRNA-mediated ZIP8 suppression (Fig. 3E, F). Conversely, transient transfection of KYSE70 cells with a ZIP8-encoding plasmid resulted in successful protein overexpression (Fig. 3G), which translated to markedly increased cell proliferation and enhanced colony formation capabilities, as evidenced by comprehensive CCK-8, foci-formation, and anchorage-independent growth assays (Fig. 3H–L). Collectively, these comprehensive investigations provide solid evidence that ZIP8 functions as a critical oncogenic driver in ESCC.

### Knockdown of ZIP8 enhances ferroptosis in ESCC cells

**Fig. 4:**
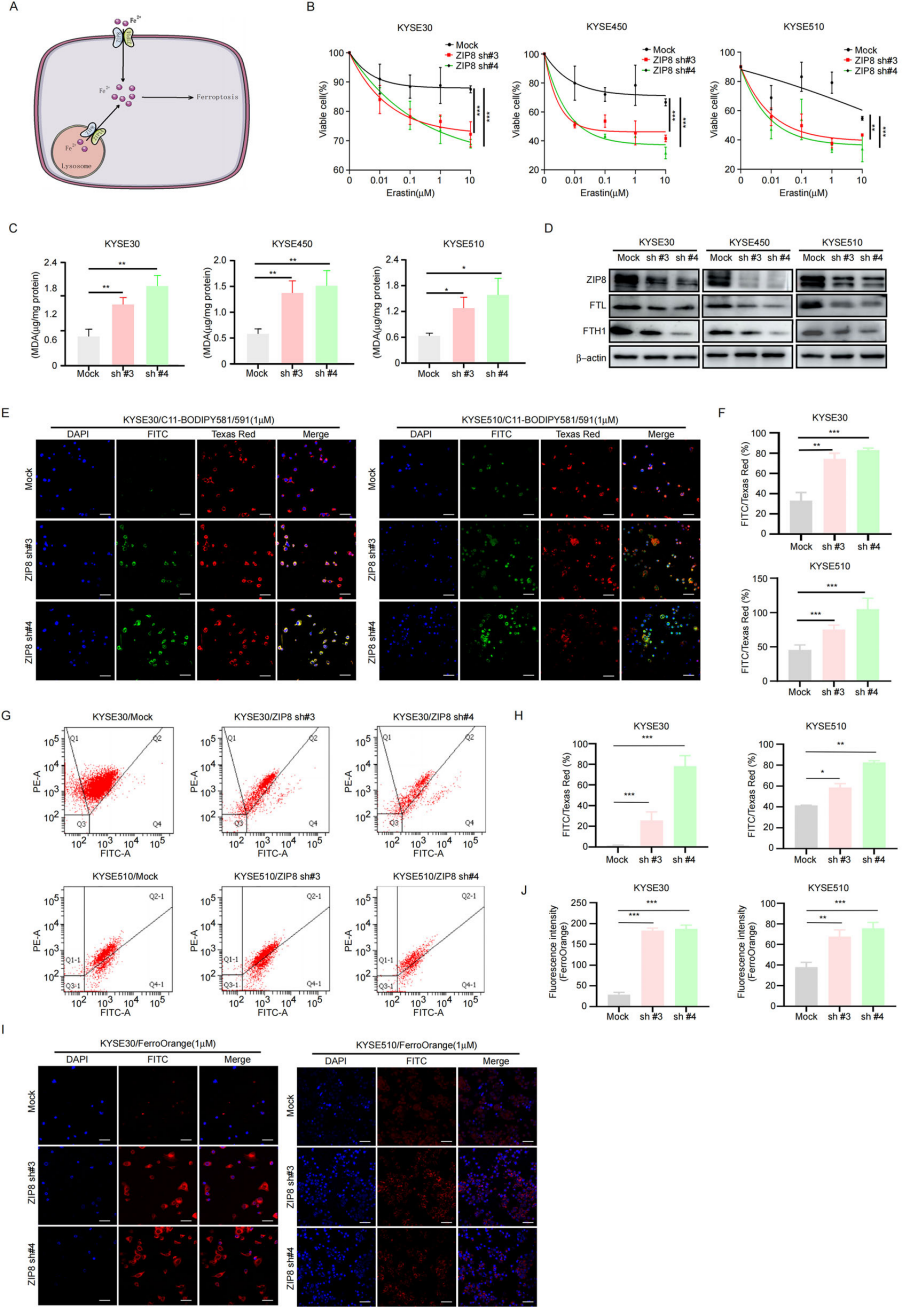
Knockdown of ZIP8 enhances ferroptosis in ESCC cells. **A** Functional schematic illustrating the role of ZIP8 in the ferroptosis pathway. **B** Sensitivity of ESCC cells to the ferroptosis activator Erastin, assessed by CCK-8 assay following treatment with increasing concentrations of Erastin (0 µM, 0.01 µM, 0.1 µM, 1 µM, 10 µM). Data represent mean ± SD of *n* = 3 independent experiments. **C** Lipid peroxidation assay to evaluate oxidative damage, with malondialdehyde (MDA) concentration measured using an MDA assay kit. **D** Western blot analysis of FTL and FTH1 expression in ESCC cells following ZIP8 knockdown. **E**, **F** Detection of lipid peroxidation in ZIP8 knockdown cells using the C11-BODIPY fluorescent probe. Scale bar, 20 µm. **G**, **H** Assessment of lipid peroxidation in KYSE30 and KYSE510 cells using the BD FACSAria II flow cytometer with the BODIPY™ 581/591 C11 probe. **I**, **J** Measurement of intracellular ferrous iron (Fe^2+^) concentration in ZIP8 knockdown cells using the FerroOrange fluorescent probe. Scale bar, 20 µm. Student’s unpaired t-test was employed for statistical analysis in (**B**, **C**, **F**, **H**, **J**). *p < 0.05, **p < 0.01, ***p < 0.001 (*n* = 3, independent experiments). Data are represented as mean ± SD.

Previous studies demonstrated that ZIP8 and ZIP14 form a complex that facilitates ferrous ion transport from lysosomes to the cytoplasm, thereby increasing intracellular ferrous ion concentrations and potentially promoting ferroptosis (Fig. 4A) [34]. To validate the impact of ZIP8 on ferroptosis in ESCC cells, we employed RNA interference to knock down ZIP8, followed by intervention with the ferroptosis inducer Erastin in ESCC cells. The results demonstrated that knocking down ZIP8 increased the sensitivity of cells to the ferroptosis inducer (Fig. 4B). Moreover, lipid peroxidation analysis revealed an increase in lipid peroxidation upon ZIP8 knockdown (Fig. 4C). Additionally, we assessed the expression of the ferroptosis markers FTL and FTH1 after ZIP8 knockdown and observed a decrease in their levels (Fig. 4D). Furthermore, utilizing the fluorescent probe BODIPY 581/591 C11 to assess lipid peroxidation, our analysis revealed that ZIP8 knockdown significantly increased lipid peroxidation levels (Fig. 4E, F). Additionally, we utilized the BD FACSAria II flow cytometer with the BODIPY™ 581/591 C11 probe to evaluate lipid peroxidation in cells subjected to ZIP8 knockdown. The flow cytometry plot (Fig. 4G) illustrate an observed increase in the FITC/Texas Red fluorescence ratio (%) in the ZIP8 knockdown groups, which is further confirmed by the statistical graph (Fig. 4H). The analysis revealed that the ZIP8 knockdown groups displayed a significant increase in FITC fluorescence intensity compared to the Texas Red fluorescence in the mock group, indicative of enhanced lipid peroxidation. To further confirm the induction of ferroptosis by ZIP8 knockdown in ESCC cells, we utilized the fluorescent probe FerroOrange and found an increase in the levels of ferrous ions (Fe^2+^) (Fig. 4I, J). In summary, our results showed that knocking ZIP8 down promotes ferroptosis in ESCC cells.

### ZIP8 enhances Zn^2+^-mediated GPX4 protein level to inhibit ferroptosis

**Fig. 5:**
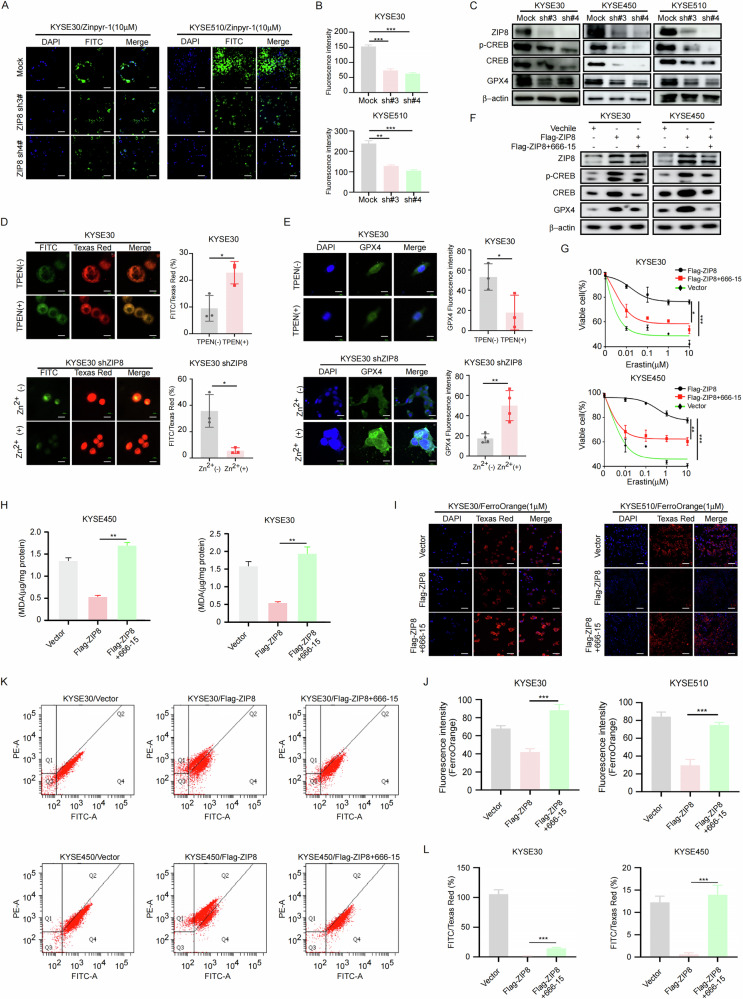
ZIP8 enhances Zn^2+^-mediated GPX4 protein levels to inhibit ferroptosis. **A**, **B** Detection of intracellular zinc ion concentration in ZIP8-knockdown ESCC cells using the fluorescent probe Zinpyr-1. Scale bar, 20 µm. **C** Western blot analysis showing the expression of GPX4 in ZIP8 knock-down ESCC cells. **D** Changes in lipid peroxidation in esophageal cancer cells KYSE30 under zinc deficiency and overload conditions. Upper: Zinc deficiency-induced lipid peroxidation using 10 µM TPEN for 2 h. Lower: Zinc overload-modulated lipid peroxidation using 10 µM zinc sulfate for 5 h in ZIP8 knockdown cells. Immunofluorescence images were captured using a confocal microscope with BODIPY™ 581/591 C11 probe. Scale bar, 20 µm. **E** Immunofluorescence analyzed the GPX4 expression level in zinc deficiency and overload conditions in esophageal cancer cells KYSE30. Upper: Zinc deficiency-induced GPX4 changes using 10 µM TPEN for 2 h. Lower: Zinc overload-modulated GPX4 changes using 0.5 mM zinc sulfate for 5 h in ZIP8 knockdown cells. Scale bar, 20 µm. **F** Western blot showing changes in ZIP8-overexpressing ESCC cells with the CREB inhibitor 666-15. **G** Sensitivity of ZIP8-overexpressing ESCC cells with the CREB inhibitor 666-15 to the ferroptosis activator Erastin. Cell viability was assessed by CCK-8 assay. **H** Lipid peroxidation analysis of ESCC after treating with the CREB inhibitor under ZIP8 upregulation. **I**, **J** Measurement of intracellular ferrous iron (Fe^2+^) concentration using FerroOrange in cells overexpressing ZIP8 with the CREB inhibitor 666-15. Scale bar, 20 µm. **K**, **L** Assessment of lipid peroxidation in ZIP8 overexpressed or 666-15 treated KYSE30 and KYSE450 cells. Cells were treated with the CREB inhibitor 666-15 at a concentration of 5 µM for 5 h and assessed using the BD FACSAria II flow cytometer with the BODIPY™ 581/591 C11 probe. Statistical analysis was performed using Student’s unpaired t test (**B**, **D**, **E**, **G**, **H**, **J**, **L**). *p < 0.05, **p < 0.01, ***p < 0.001 (*n* = 3, inde*p*endent experiments). Data are represented as mean ± SD.

While ZIP8 is a well-established regulator of ferroptosis [35], the precise role that its heightened expression in esophageal cancer plays within the ferroptosis pathway remains unclear. To assess the functional role of ZIP8 as a zinc transporter, we evaluated intracellular zinc concentrations using con-focal fluorescence microscopy. Immunofluorescence analysis demonstrated that ZIP8 knockdown resulted in a significant reduction in intracellular zinc levels (Fig. 5A, B). Previous research has demonstrated that pancreatic cancer cells overexpressing ZIP4 show increased CREB phosphorylation due to elevated zinc levels, with CREB activation notably suppressed in ASPC-1 cells with ZIP4 silencing [36]. Similarly, ZIP10 can initiate CREB phosphorylation, activate the PI3K/AKT signaling pathway via ITGA10, and promote osteosarcoma cell proliferation and drug resistance [37]. These findings collectively suggest that CREB phosphorylation is zinc ion-dependent. In our study, ZIP8 knockdown resulted in decreased CREB phosphorylation and reduced expression of the ferroptosis inhibitor GPX4, and reduced the total CREB protein level (Fig. 5C). To investigate the relationship between CREB phosphorylation status and protein stability, we transfected KYSE450 and KYSE510 cells with wild-type CREB (CREB-WT) or phosphorylation mutants (CREB-S133A and CREB-S133D) plasmids, followed by cycloheximide (CHX) treatment. The CREB-S133A mutant demonstrated significantly accelerated degradation compared to both CREB-WT and CREB-S133D, suggesting that phosphorylation at serine 133 plays a crucial role in regulating CREB protein stability (Fig. S1A). To investigate the impact of zinc ions on ferroptosis, we used the zinc chelator (PTEN) to remove zinc ions and added zinc sulfate (ZnSO_2_) to increase zinc ion concentration to verify the effect of zinc ion concentration on GPX4 and ferroptosis. The results indicated that Zinc ion depletion inhibits GPX4 expression and promotes ferroptosis, and Zinc supplementation in ZIP8 knockdown cells enhances GPX4 expression and confers resistance to ferroptosis (Figs. 5D, E, S1B, C). In summary, zinc ions critically modulate CREB phosphorylation and GPX4 expression, with ZIP8 emerging as a key regulatory factor in zinc-dependent CREB phosphorylation within ESCC.

As reported, CREB can suppress lipid peroxidation by binding to the promoter region of glutathione peroxidase 4 (GPX4), suggesting that CREB upregulates the transcriptional level of GPX4 and inhibits the ferroptosis pathway in lung adenocarcinoma [38]. We propose a hypothesis that the ZIP8-pCREB-GPX4 axis can regulate the ferroptosis pathway in ESCC cells. To further support our hypothesis, we performed experiments using CREB inhibitor (666-15) in ZIP8-overexpressing ESCC cell lines (KYSE30 and KYSE450). The results demonstrated that 666-15 in ZIP8-overexpressing cells significantly reduced the protein level of GPX4 (Fig. 5F). To further investigate the function of ZIP8-pCREB-GPX4 axis in ESCC, we conducted CCK-8 proliferation assays. Our results demonstrated that ZIP8 overexpression reduced cell sensitivity to Erastin, while treatment with 666-15 effectively restored the cells’ sensitivity to the drug. Furthermore, lipid peroxidation analysis showed an elevation in lipid peroxidation upon 666-15 (Fig. 5G, H). Similarly, using the fluorescent probe FerroOrange, we detected a significant increase in the concentration of iron ions in the cytoplasm upon 666-15 in ZIP8 overexpression cells (Fig. 5I, J). Furthermore, we employed the BD FACSAria II flow cytometer equipped with the BODIPY™ 581/591 C11 probe to assess lipid peroxidation in ZIP8-overexpressing cells treated with 666-15. Flow cytometry analysis revealed a significant increase in the FITC/Texas Red fluorescence ratio (%) following 666-15 treatment (Fig. 5K, L). This analysis indicated that the group treated with 666-15 exhibited a marked increase in FITC fluorescence intensity relative to Texas Red fluorescence in the control group, suggesting an augmentation in lipid peroxidation. In summary, all the experimental results have confirmed that high expression of ZIP8 increases Zn^2+^-dependent phosphorylation levels of CREB, thereby enhancing the protein level of GPX4 and inhibiting ferroptosis.

### Nobiletin is a potent ZIP8 inhibitor

**Fig. 6:**
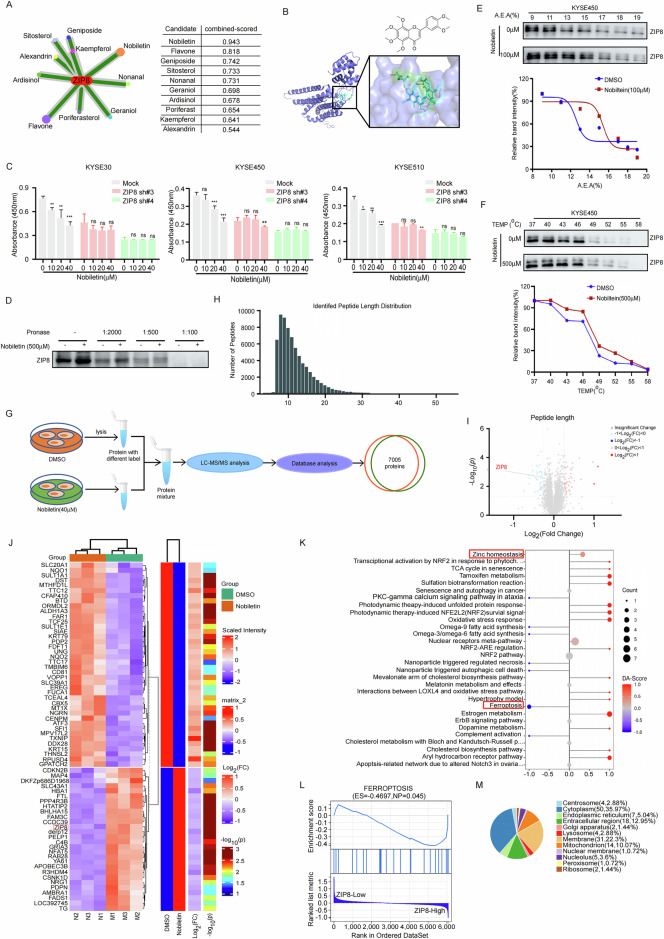
Nobiletin is a potent ZIP8 inhibitor. **A** Analysis of the cancer HSP database to identify potential interactions between ZIP8 and traditional chinese medicine compounds. **B** Computational modeling depicting the binding interface between Nobiletin and ZIP8. **C** ZIP8 knockdown cell lines KYSE30, KYSE450, and KYSE510 were treated with increasing concentrations of Nobiletin (0 μM, 10 μM, 20 μM, 40 μM) to assess cell proliferation using the CCK-8 assay. Statistical analysis was performed using Student’s unpaired t test. *p < 0.05, **p < 0.01, ***p < 0.001 (*n* = 3, inde*p*endent experiments). Data are represented as mean ± SD. **D** Drug affinity responsive target stability (DARTS) assay for ZIP8 identification in KYSE450 cells. **E** Solvent-induced protein precipitation (SIP) assay to assess the binding affinity of Nobiletin to ZIP8 in KYSE450 cells. **F** CETSA assay showing the binding affinity of Nobiletin to ZIP8 in KYSE450 cells. **G** Schematic diagram outlining the process of quantitative proteomics analysis. KYSE450 cells were treated with DMSO or Nobiletin (40 µM) for 24 h, followed by LC-MS/MS analysis. **H** Distribution of peptide length for the 7005 proteins identified. Peptide length is plotted on the X axis, and the number of peptides on the Y axis. **I** Volcano plot illustrating differential protein expression. **J** Heatmap representation of differentially expressed proteins between DMSO and Nobiletin (40 µM) groups. **K** Wikipathway enrichment analysis of differentially expressed proteins. **L** GSEA analysis of ZIP8’s role in ferroptosis based on proteomic analysis. **M** Sub-cellular localization of differentially expressed proteins.

In order to identify the small molecule inhibitors targeting ZIP8, we used database CancerHSP, which is a database of traditional Chinese medicine that captures the relationship between drugs, targets and diseases. By collecting the data set predicted by the drug-protein interaction in the database, the data set is preprocessed (Fig. 6A, left panel). The protein sequence and the protein characteristics are extracted. Based on the protein characteristics, the molecular mechanism of drugs and proteins is encoded at the same time, and the probability score of drug-protein pair interaction is calculated according to the coding (Fig. 6A, right panel). In addition, computational model was used to illustrate the bind between ZIP8 and Nobiletin, which showed that Nobiletin binds to ZIP8 at GLU-65 and ARG-116, and the chemical structure of Nobiletin shown in Fig. 6B. In order to verify the effect of Nobiletin on ZIP8 in ESCC cells, we knocked down ZIP8 in KYSE30, KYSE450, and KYSE510 cell lines and treated them with different concentrations of Nobiletin (0 µM, 10 µM, 20 µM, and 40 µM). The results indicated that Nobiletin had no effect on ZIP8 knockdown cells (Fig. 6C), suggesting ZIP8 as the specific target of Nobiletin. Moreover, the DARTS assay was conducted to confirm that ZIP8 is a direct target of Nobiletin. Upon the addition of pronase, Nobiletin demonstrated the ability to protect ZIP8 from proteolytic degradation. These findings collectively provide strong evidence that ZIP8 is indeed the target of Nobiletin (Fig. 6D). To further confirm the binding interaction, we conducted a solvent-induced protein precipitation (SIP) assay with cell lysates and varying concentrations of A.E.A. solution (50% acetone, 50% ethanol, and 0.1% acetic acid). The results revealed that the dissociation rate of ZIP8, when incubated with Nobiletin, was accelerated compared to the control. This finding hints at Nobiletin’s ability to bind to ZIP8, potentially influencing its stability during the SIP assay (Fig. 6E). Additionally, the Cellular Thermal Shift Assay (CETSAs) demonstrated the binding affinity of Nobiletin to ZIP8 in ESCC cells (Fig. 6F). The results revealed that the dissociation rate of ZIP8 incubated with Nobiletin was faster compared to the control condition. This observation suggests that ZIP8 is the potent target of Nobiletin.

Proteomic analysis was employed to delve deeper into the underlying mechanisms of Nobiletin treatment and to provide a comprehensive assessment of changes in protein levels post-treatment. Our proteomics approach successfully identified and analyzed 7005 proteins with coverage and overlap. The flowchart and quality control profile for this analysis are depicted in Fig. 6G, H. To identify differentially expressed proteins, we set a threshold of 1.2-fold change from baseline, with a t-test *p*-value less than 0.05 as the standard. Then, 46 proteins were up-regulated while 35 proteins were down-regulated among these differentially expressed proteins (Fig. 6I, J).

The KEGG pathway enrichment analysis identified the top 30 signaling pathways, highlighting both upregulated and downregulated pathways following Nobiletin treatment. Among these, the upstream pathway exhibiting the most significant alteration was related to zinc homeostasis, underscoring the pronounced effect of nobiletin on zinc regulation. Conversely, the downstream pathway most prominently affected involved ferroptosis, specifically iron-induced cell death (Fig. 6K). Proteomic analysis further revealed that changes in protein expression were closely linked to ZIP8’s role in ferroptosis (Table S1), with high ZIP8 expression showing a negative correlation with ferroptosis induction (Fig. 6L). Subcellular localization analysis of differentially expressed proteins indicated that 35.97% were localized in the cytoplasm, and 22.3% in the membrane (Fig. 6M). These findings suggest that the regulation of zinc homeostasis and the activation of ferroptosis may constitute key mechanisms underlying the anti-tumor activity of Nobiletin.

### Nobiletin promotes ferroptosis in ESCC cells

**Fig. 7:**
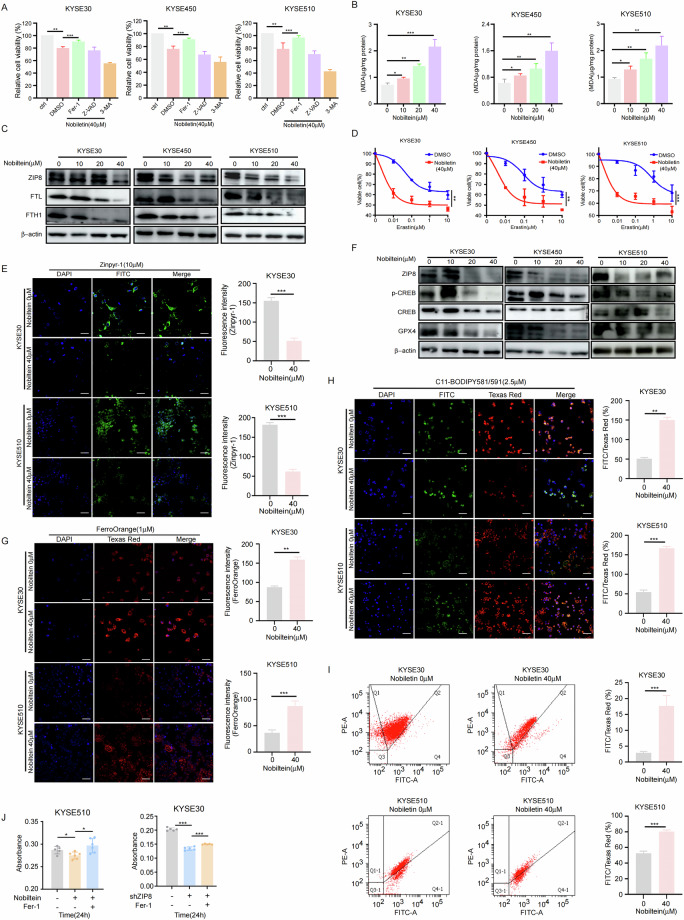
Nobiletin promotes ferroptosis in ESCC cells. **A** CCK-8 assay showing the effect of Nobiletin treatment on ESCC cells pretreated with DMSO and various inhibitors (Fer-1, Z-VAD-FMK, 3-MA) for 24 h. **B** Lipid peroxidation levels, indicated by malondialdehyde (MDA) concentration, measured using an MDA assay kit after treatment with different concentrations of Nobiletin (0 μM, 10 μM, 20 μM, 40 μM). **C** Western blot analysis of FTL and FTH1 expression in ESCC cells treated with increasing concentrations of Nobiletin (0 μM, 10 μM, 20 μM, 40 μM). **D** Cells were treated with DMSO or Nobiletin (40 μM), followed by the addition of increasing concentrations of erastin (0 μM, 0.01 μM, 0.1 μM, 1 μM, 10 μM). Cell proliferation was assessed using the CCK-8 assay. **E** Intracellular zinc ion concentration in ESCC cells treated with Nobiletin, measured using the fluorescent probe Zinpyr-1. Scale bar, 20 μm. **F** Western blot analysis of p-CREB, CREB, and GPX4 expression in ESCC cells treated with different concentrations of Nobiletin. **G** Intracellular ferrous iron (Fe^2+^) concentration measured using FerroOrange in cells treated with different concentrations of Nobiletin. Scale bar, 20 μm. **H** Lipid peroxidation levels measured using the fluorescent probe C11-BODIPY in cells treated with different concentrations of Nobiletin. Scale bar, 20 μm. **I** Assessment of lipid peroxidation in KYSE30 and KYSE510 cells using the BD FACSAria II flow cytometer with the BODIPY™ 581/591 C11 probe after treatment with Nobiletin for 24 h. **J** Cell proliferation was assessed using the CCK-8 assay following treatment with ferrostatin-1 (Fer-1) in Nobiletin-treated or ZIP8 knockdown cells. *n* = 3, independent experiments. Student’s unpaired *t* test was used for statistical analysis (**A**, **B**, **D**, **E**, **G**, **H**, **I**, J). *p < 0.05, **p < 0.01, ***p < 0.001. Data are re*p*resented as mean ± SD.

To validate whether the suppression of ESCC cell growth by Nobiletin is mediated through the ferroptosis pathway, we treated Nobiletin-exposed ESCC cells with the ferroptosis inhibitor Fer-1. Simultaneously, the apoptosis inhibitor Z-VAD and the autophagy inhibitor 3-MA were utilized as controls. The findings indicated that nobiletin inhibited ESCC cell growth primarily via the ferroptosis pathway, rather than through mechanisms involving apoptosis or autophagy (Fig. 7A). Additionally, by examining lipid peroxidation, we observed a dose-dependent increase in lipid peroxidation in response to Nobiletin treatment (Fig. 7B). Furthermore, we assessed the expression level of ferritin, FTL and FTH1, as markers of ferroptosis, and observed a decrease in FTL and FTH1 levels upon Nobiletin treatment (Fig. 7C), indicating the occurrence of ferroptosis. Cells treated with Nobiletin exhibited increased sensitivity to the ferroptosis inducer Erastin (Fig. 7D). To confirm that Nobiletin targets ZIP8 to regulate GPX4 in a zinc-dependent manner, thereby modulating ferroptosis in ESCC cells, we first employed the Zinpyr-1 fluorescent probe to evaluate changes in cellular zinc ion content. The results revealed a reduction in the fluorescence intensity of the Zinpyr-1 probe following Nobiletin treatment (Fig. 7E). Subsequently, Western blot analysis of Nobiletin-treated ESCC cells demonstrated a decrease in the expression levels of p-CREB and GPX4 (Fig. 7F). To further validate the induction of ferroptosis by Nobiletin, we utilized the FerroOrange fluorescent probe, which indicated an increase in ferrous iron levels (Fig. 7G). Additionally, using the BODIPY 581/591 C11 probe, we observed elevated Lipid peroxidation levels in both the cytoplasm and cell membrane (Fig. 7H). Lipid peroxidation was further assessed by flow cytometry using the BD FACSAria II and the BODIPY™ 581/591 C11 probe, which confirmed increased lipid peroxidation in Nobiletin-treated cells (Fig. 7I). To further conformed the ferroptosis pathway in ESCC progression, ferroptosis inhibitor was used to treat ZIP8-knockdown cells and Nobiletin-treated cells. The results revealed that ferroptosis inhibitor treatment rescued cell growth in both ZIP8-knockdown cells and nobiletin-treated cells (Fig. 7J). These findings provide compelling evidence supporting the hypothesis that Nobiletin targets ZIP8 to reduce zinc-dependent GPX4 levels, thereby promoting ferroptosis in ESCC cells.

### Nobiletin suppresses cell growth of ESCC in vitro and in vivo

**Fig. 8:**
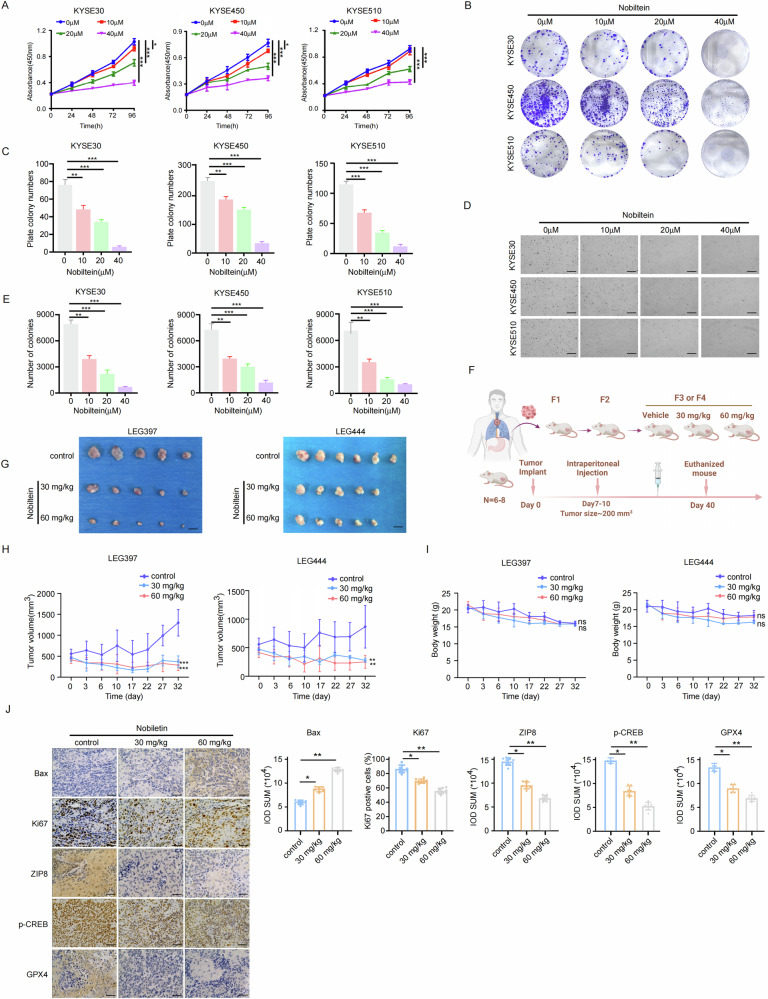
Nobiletin suppresses cell growth of ESCC in vitro and in vivo. **A** Proliferation of KYSE30, KYSE450, and KYSE510 cells measured by CCK-8 assays at 24 h, 48 h, 72 h, and 96 h intervals. **B**, **C** Colony formation assays and crystal violet staining to assess the proliferation abilities of KYSE30, KYSE450, and KYSE510 cells. **D**, **E** Anchorage-independent cell growth assay to evaluate the effect of Nobiletin (0 μM, 10 μM, 20 μM, 40 μM) on cell growth (scale bar, 200 μm). **F** Schematic diagram of the patient-derived xenograft (PDX) model. **G**, **H** Tumor size and volume measurements in the PDX model following Nobiletin treatment. **I** Body weights of mice across different treatment groups. **J** IHC staining for BAX, Ki67, ZIP8, p-CREB and GPX4 on PDX mouse tissues. IOD SUM represents the integrated optical density value of statistics. Data presented as mean ± S.D. from three independent experiments. Statistical significance was assessed using Student’s unpaired t-test (**A**, **C**, **E**, **H**, **I**, **J**). *p < 0.05, **p < 0.01, **p < 0.001.

To evaluate the effects of Nobiletin on ESCC cells, varying concentrations of Nobiletin (0–40 μM) were uesd. The results indicated that Nobiletin inhibits cell proliferation in a dose-dependent manner (Fig. 8A). Additionally, Nobiletin treatment dose-dependently diminished the colony formation capacity of ESCC cells (Fig. 8B, C). Moreover, the soft agar experiment further validated the inhibitory effect of Nobiletin on ESCC cell growth (Fig. 8D, E). To further analyzed the effects of Nobiletin on ESCC, we utilized a PDX mouse model (Fig. 8F) to assess its anti-tumor efficacy in vivo. Compared to the control group, Nobiletin treatment resulted in a significant reduction in tumor volume (Fig. 8G, H), with no substantial change in the body weights of mice across the different treatment groups (Fig. 8I). Additionally, immunohistochemistry (IHC) analysis of PDX mouse tissues revealed that Nobiletin treatment led to decreased expression of Ki-67, ZIP8, p-CREB, and GPX4, alongside an increase in the apoptosis marker Bax (Fig. 8J). Collectively, these results suggested that Nobiletin effectively inhibits ESCC growth both in vivo and in vitro.

## Discussion

Esophageal cancer is the ninth most common cancer worldwide and the sixth leading cause of cancer deaths, with Eastern Asia having the highest incidence [1]. Nevertheless, the underlying mechanism of esophageal cancer remains largely elusive. In this study, we confirmed that ZIP8 was highly expressed in ESCC cells and tissues, which promoted the growth of ESCC cells and inhibit ferroptosis. These findings indicated that ZIP8 is the key regulator of esophageal cancer and can be used as the target of target therapy.

**Fig. 9:**
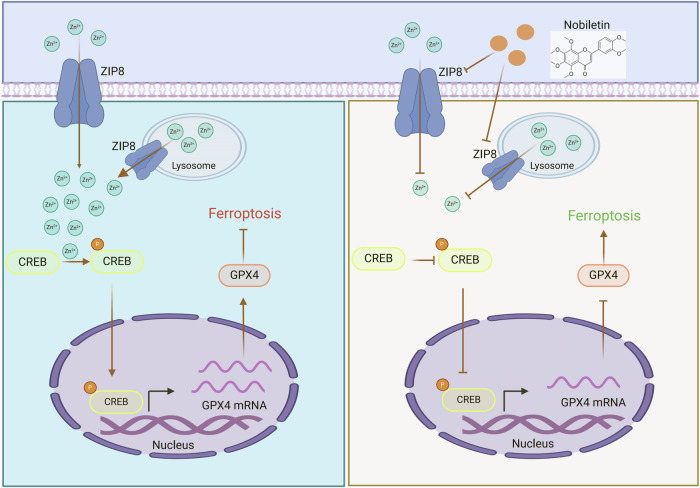
The working model illustrating the role of ZIP8 in modulating ferroptosis in ESCC cells through the ZIP8-pCREB-GPX4 axis.

ZIP8 has been reported to promote cell growth and survival in cancer. Knockdown of zinc transporter ZIP8 expression inhibits neuroblastoma progression and metastasis in vitro [13]. Presently, research into zinc homeostasis within the context of esophageal cancer remains sparse. Our experimental findings indicate a significant association between zinc homeostasis and the initiation and progression of esophageal cancer. High expression of ZIP8 increases Zn^2+^-dependent phosphorylation levels of CREB, thereby enhancing the protein level of GPX4 and inhibiting ferroptosis (Fig. 9). Moreover, the proteomic sequencing analysis coupled with rigorous experimental validation underscores the pivotal role of zinc ions in triggering ferroptosis. Our findings highlight the criticality of zinc homeostasis and ferroptosis mechanisms, reinforcing the essential function of ZIP8 in modulating the ferroptosis pathway.

Currently, the activity and potential mechanism of natural products have been important for drug discovery and development. Fruits, which are natural products, could have an important role in the prevention of cancer [39–41]. Nobiletin is a major component of polymethoxylated flavones in the citrus fruit peel. In recent years, the antitumor activity of Nobiletin has gradually received attention [28]. Previous studies reported the anti-cancer effects of Nobiletin. Nobiletin inhibits de novo FA synthesis to alleviate gastric cancer progression by regulating endoplasmic reticulum stress [42]. Nobiletin inhibits breast cancer cell migration and invasion by suppressing the IL-6-induced ERK-STAT and JNK-c-JUN pathways [43]. Nobiletin Triggers Reactive Oxygen Species-Mediated Pyroptosis through Regulating Autophagy in Ovarian Cancer Cells [44]. But it’s role in ESCC is poorly understood. Our results demonstrated Nobiletin’s potent anti-proliferative effects against ESCC cells in vitro and in vivo. Interestingly, after adding Nobiletin, the protein level of ZIP8 decreased. According to reports, protein degradation is mainly regulated by two systems: the ubiquitin proteasome system or the lysosomal autophagosome system [45]. We speculate that this phenomenon may be due to the regulation of the protein degradation system of ZIP8 protein after treatment with Nobiletin, resulting in a decrease in ZIP8 protein levels. The specific degradation pathways and mechanisms still need to be studied.

In conclusion, we confirmed that ZIP8 depletion can promote ferroptosis to inhibit the growth of ESCC cells. Nobiletin has great potential as a novel lead compound for ESCC. ZIP8 does not affect ferroptosis in a single way. It can regulate ferroptosis by regulating the level of iron ions and zinc ions, which are realized by different way.

## Supplementary information

Supplementary Figure S1

Supplementary Figure S1 legend

Supplementary Table S1

original western blot images

## Data Availability

The data that support the findings of this study are available from the corresponding author upon reasonable request.
