# Supplementary figures and images for: ZIP8 modulates ferroptosis to drive esophageal carcinoma progression
Source: Cell Death Dis. 2025 May 6;16(1):366. doi: 10.1038/s41419-025-07692-z (PMC12056185; doi:10.1038/s41419-025-07692-z)

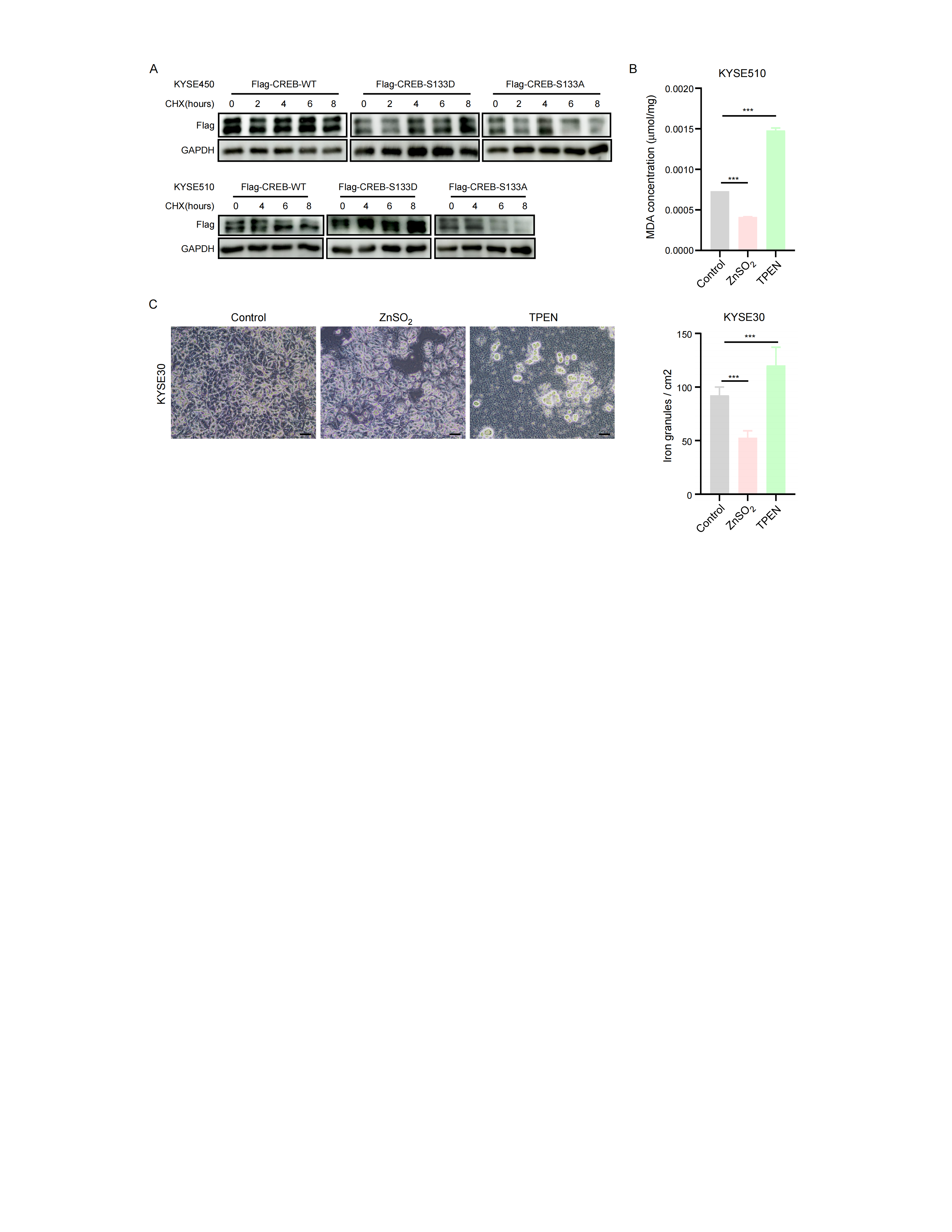

Supplement: Supplementary file 1 — Supplementary Figure S1 [file 41419_2025_7692_MOESM1_ESM.tif]
