# Supplementary material for: ZIP8 modulates ferroptosis to drive esophageal carcinoma progression
Source: Cell Death Dis. 2025 May 6;16(1):366. doi: 10.1038/s41419-025-07692-z (PMC12056185; doi:10.1038/s41419-025-07692-z)
Supplement: Supplementary file 2 — Supplementary Figure S1 legend [file 41419_2025_7692_MOESM2_ESM.docx]

**Fig. S1 A** The relationship between CREB phosphorylation status and protein stability in KYSE450 and KYSE510 cells. **B** Changes in MDA in esophageal cancer cells KYSE510 under zinc deficiency and overload conditions. Group control: untreated KYSE510 cells; Group ZnSO_2_: Zinc overload-induced lipid peroxidation using 10 µM zinc sulfate for 12 hours in KYSE510 cells. Group TPEN: Zinc deficiency-modulated lipid peroxidation using 10 µM TPEN for 12 hours in KYSE510 cells. **C** Prussian blue staining for the detection of iron accumulation in KYSE30 cells. Iron granules were stained yellow to yellow-brown, while the cell nuclei appeared red. Scale bar, 50 µm. Left panel: Fe^3+^ staining graphs; Right panel: Statistical plot. The Data presented as mean ± S.D. from three independent experiments. Statistical significance was assessed using Student's unpaired t-test (B). **p* < 0.05, ***p* < 0.01, ***p* < 0.001.
