# Supplementary material for: ZIP8 modulates ferroptosis to drive esophageal carcinoma progression
Source: Cell Death Dis. 2025 May 6;16(1):366. doi: 10.1038/s41419-025-07692-z (PMC12056185; doi:10.1038/s41419-025-07692-z)
Supplement: Supplementary file 3 — Supplementary Table S1 [file 41419_2025_7692_MOESM3_ESM.docx]

**Table S1** Proteomic analysis reveals the link between protein expression changes and ZIP8’s role in ferroptosis

| Tag | logFC | AveExpr | t | P.Value | adj.P.Val | B |
| --- | --- | --- | --- | --- | --- | --- |
| SLC39A8 | 1.503867333 | 7.915999 | 10.49514898 | 1.03E-11 | 1.58E-09 | 16.77240718 |
| SLC39A14 | 1.851902 | 8.613197 | 9.5961567 | 8.70E-11 | 6.62E-09 | 14.66158522 |
| SLC3A2 | 1.113694667 | 10.90527267 | 9.352062134 | 1.58E-10 | 1.05E-08 | 14.06936905 |
| PRNP | 1.253446667 | 11.66735667 | 9.250165546 | 2.04E-10 | 1.27E-08 | 13.81973647 |
| CYBB | 1.826859333 | 9.319929667 | 9.177338241 | 2.44E-10 | 1.44E-08 | 13.64045014 |
| TFRC | 1.694884667 | 10.44435767 | 8.862294275 | 5.39E-10 | 2.68E-08 | 12.85655796 |
| HMOX1 | -1.315918 | 9.978394333 | -4.500170038 | 8.99E-05 | 0.000422751 | 0.968644121 |
| SLC7A11 | 1.931748667 | 8.763833667 | 4.485535295 | 9.37E-05 | 0.000438262 | 0.928139734 |
| CP | 2.023575333 | 5.925502333 | 4.165740576 | 0.000230775 | 0.000941889 | 0.050553289 |
| GCLC | 1.024994667 | 9.604998 | 3.633757373 | 0.001001521 | 0.00327616 | -1.365999765 |
