# Supplementary material for: ZIP8 modulates ferroptosis to drive esophageal carcinoma progression
Source: Cell Death Dis. 2025 May 6;16(1):366. doi: 10.1038/s41419-025-07692-z (PMC12056185; doi:10.1038/s41419-025-07692-z)

Figue 2J-ZIP8-1

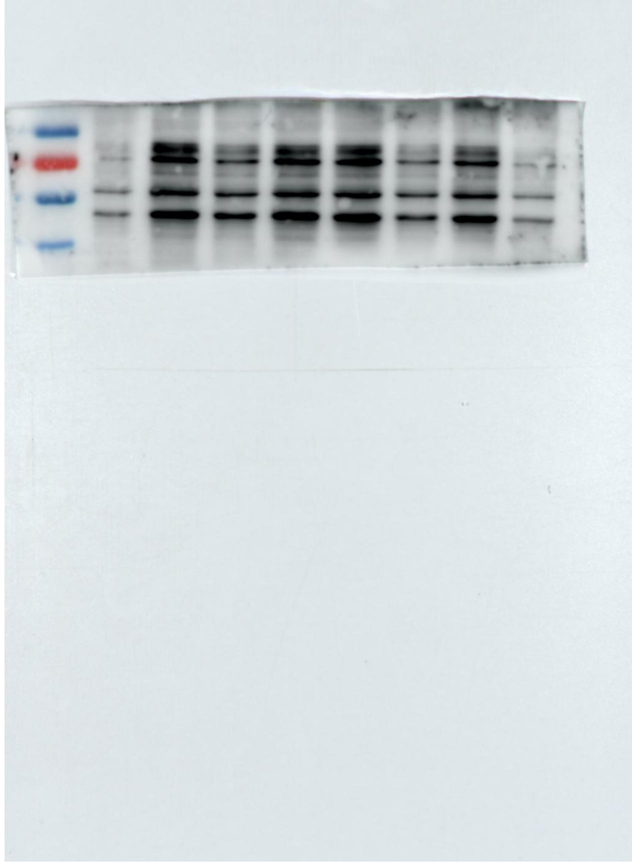

Figue 2J-actin-1

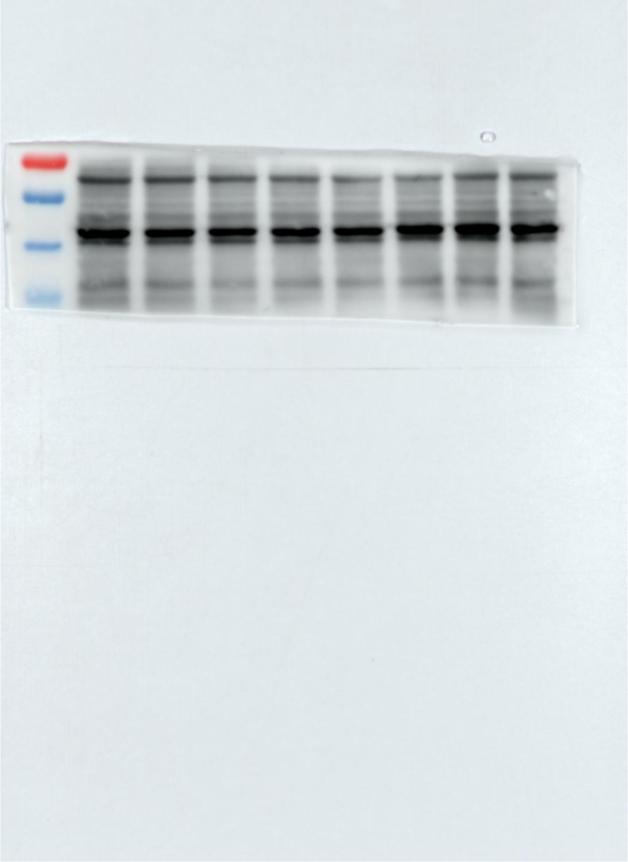

Figue 3A-ZIP8-30

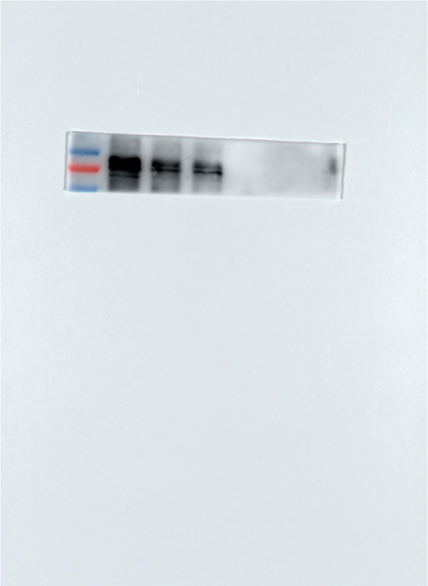

Figue 3A-ZIP8-450

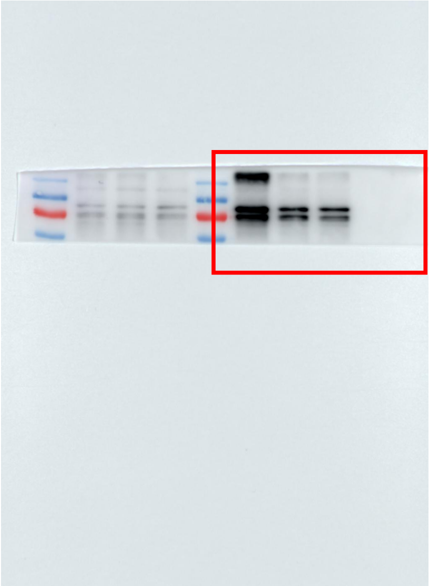

Figue 3A-ZIP8-510

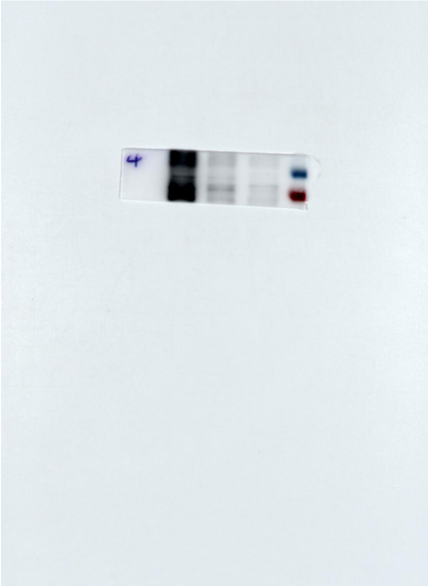

Figue 3A-actin-30

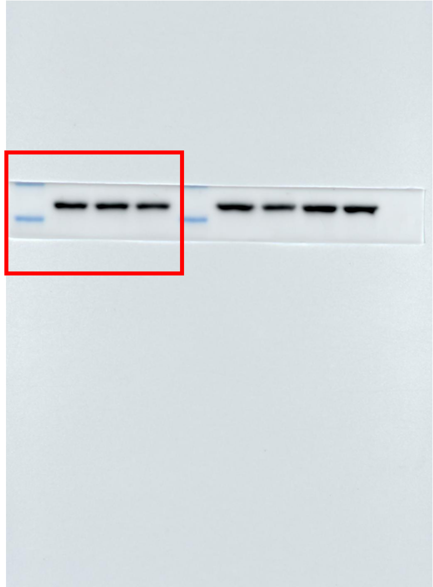

Figue 3A-actin-450

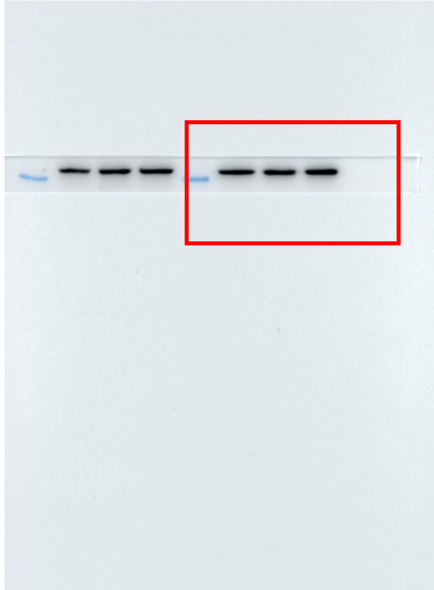

Figue 3A-actin-510

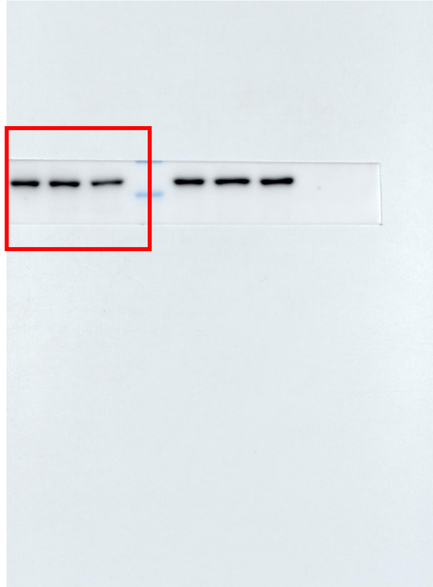

Figue 3G-ZIP70

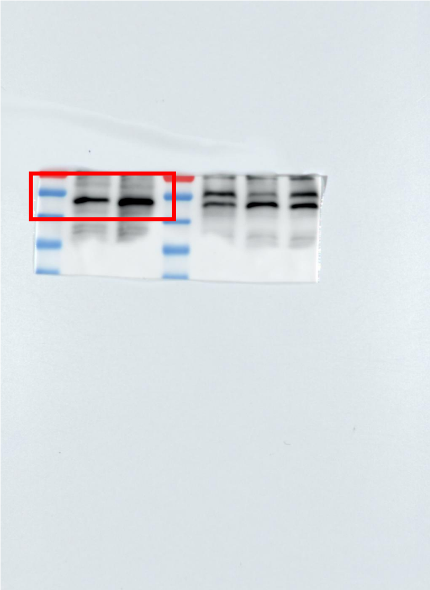

Figue 3G-actin-70

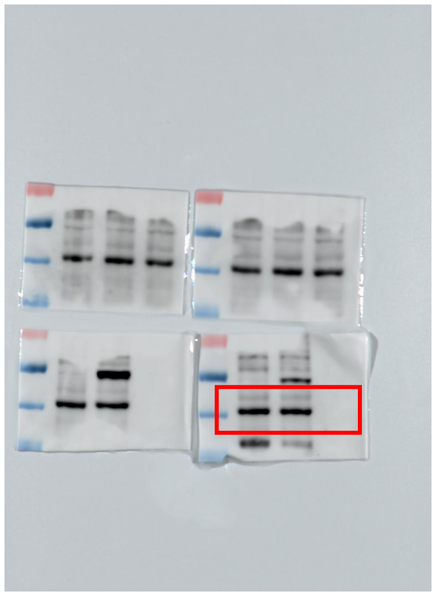

Figure 4D-30-actin

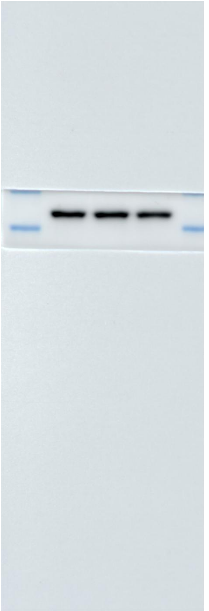

Figure 4D-30-FTH1

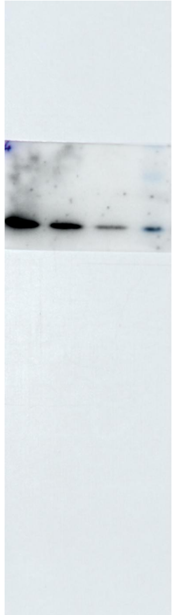

Figure 4D-30-FTL

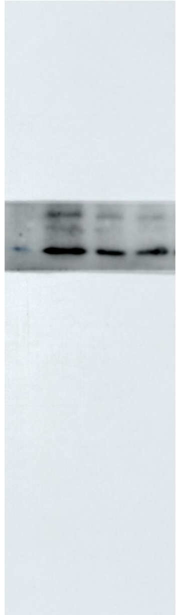

Figure 4D-30-ZIP8

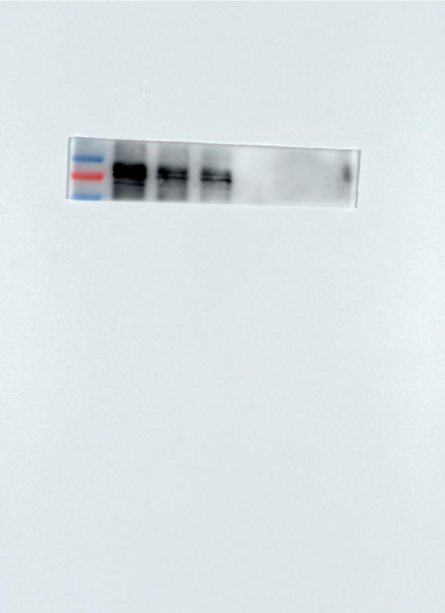

Figure 4D-450-actin

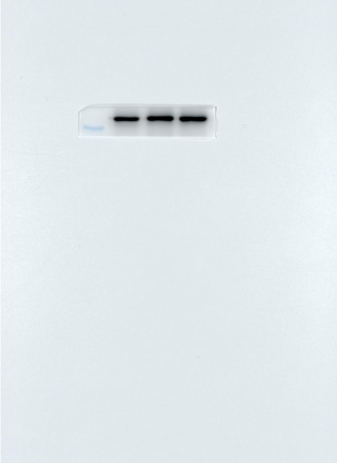

Figure 4D-450-FTH1

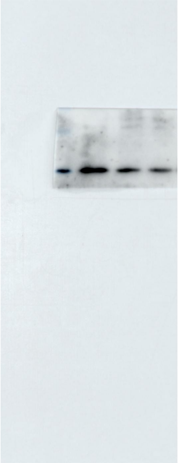

Figure 4D-450-FTL

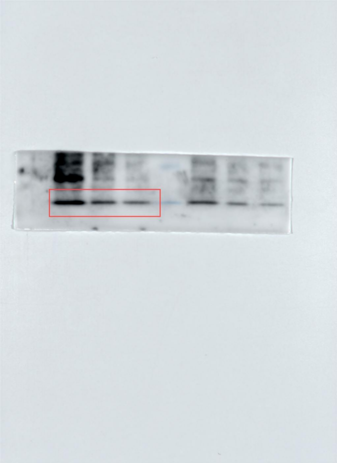

Figure 4D-450-ZIP8

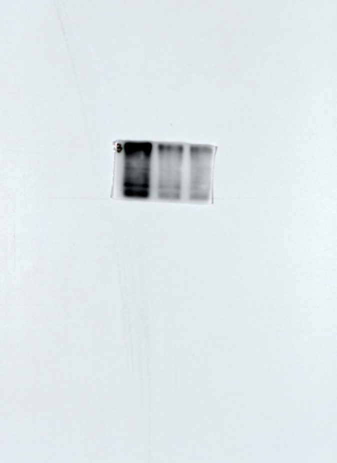

Figure 4D-510-actin

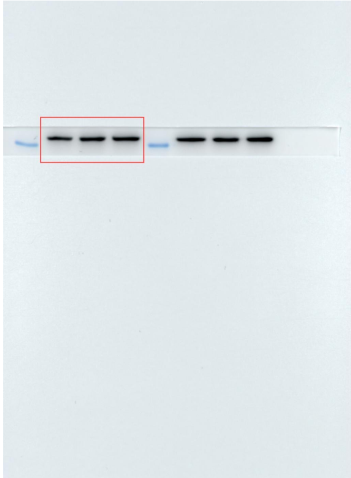

Figure 4D-510-FTH1

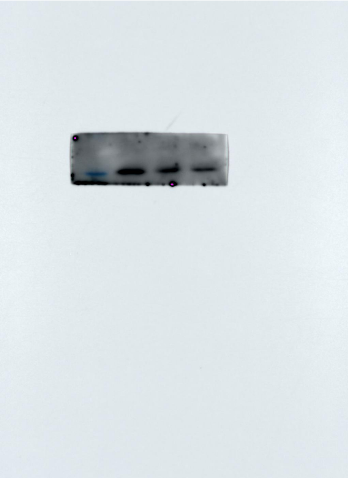

Figure 4D-510-FTL

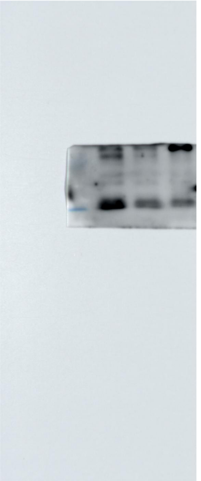

Figure 4D-510-ZIP8

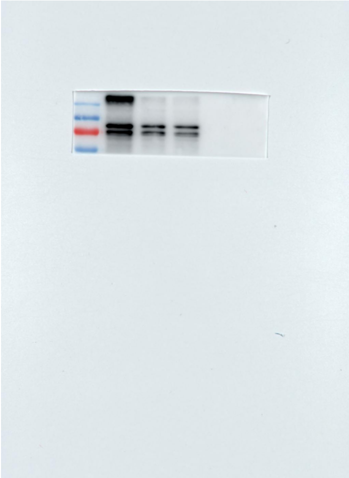

Figure 5-C30-actin

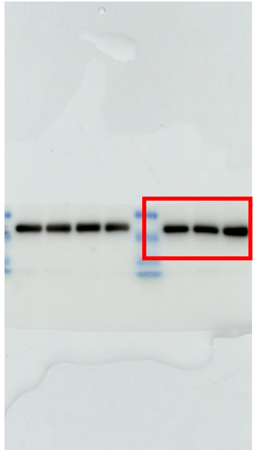

Figure 5C-30-CREB

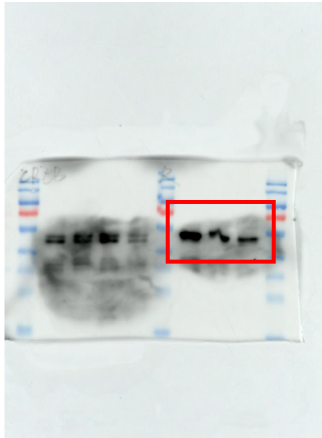

Figure 5C-30-GPX4

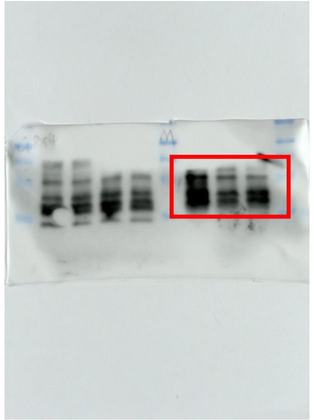

Figure 5C-30-p-CREB-1

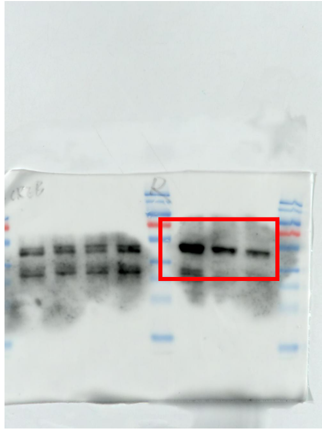

Figure 5C-30-ZIP8

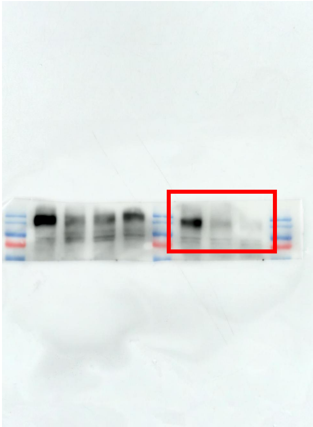

Figure 5C-450-actin

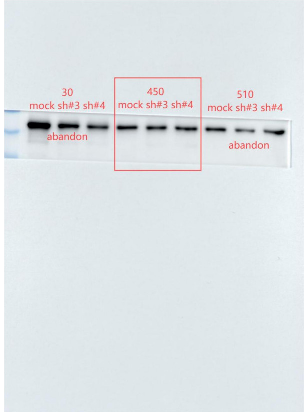

Figure 5C-450-CREB

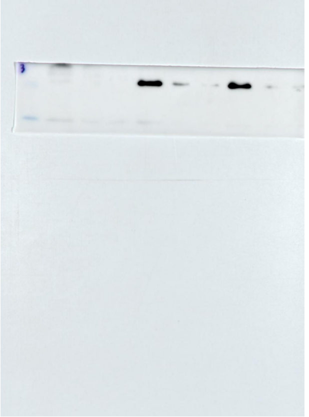

Figure 5C-450-GPX4

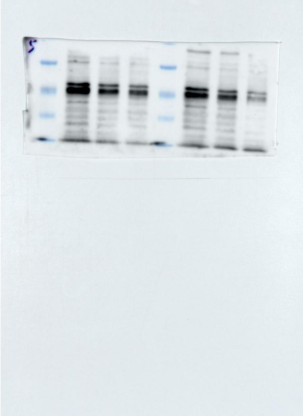

Figure 5C-450-p-CREB

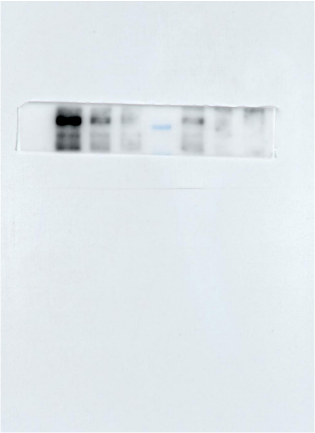

Figure 5C-450-ZIP8

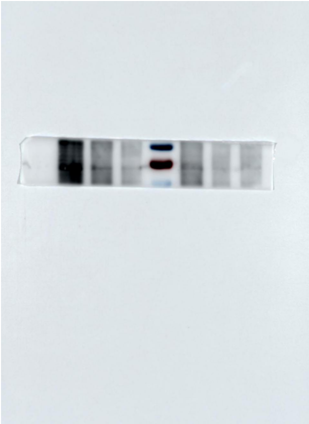

Figure 5C-510-actin

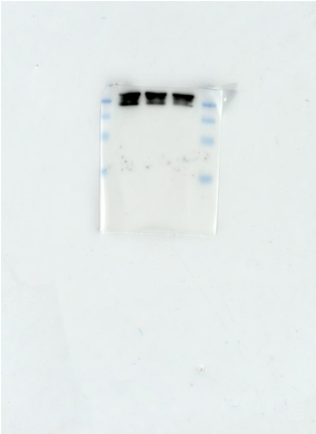

Figure 5C-510-CREB

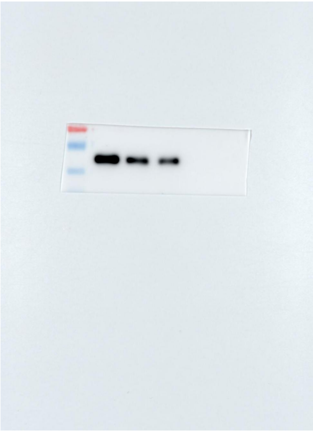

Figure 5C-510-GPX4

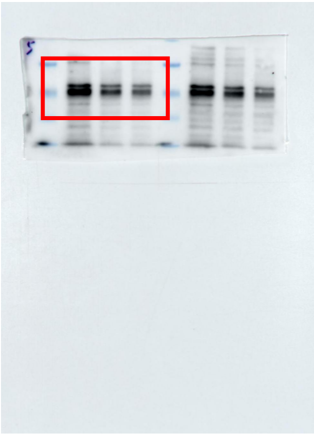

Figure 5C-510-p-CREB

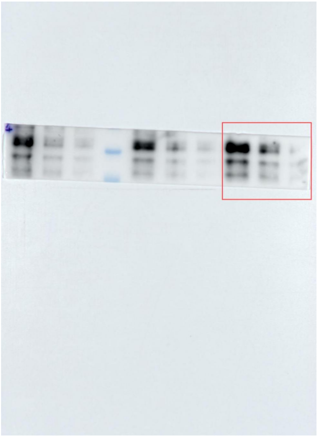

Figure 5C-510-ZIP8

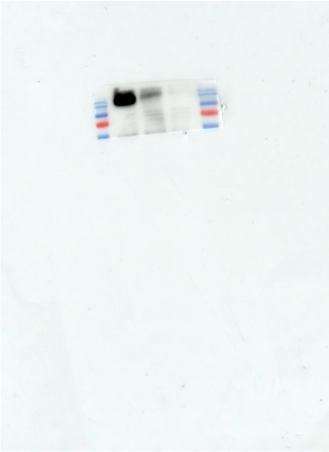

Figure 5D-30-actin

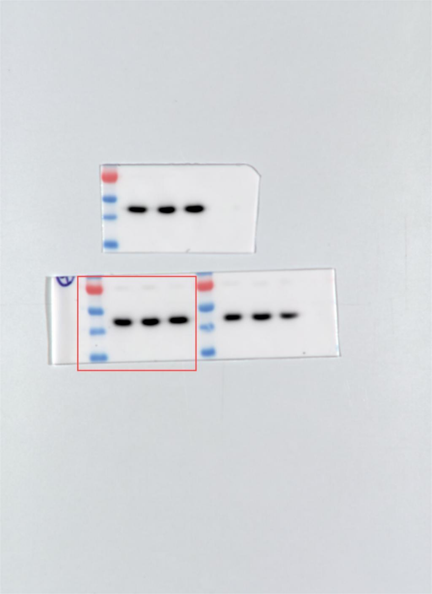

Figure 5D-30-CREB

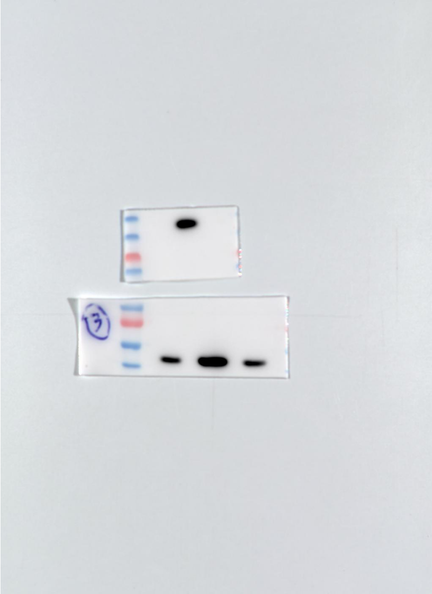

Figure 5D-30-GPX4

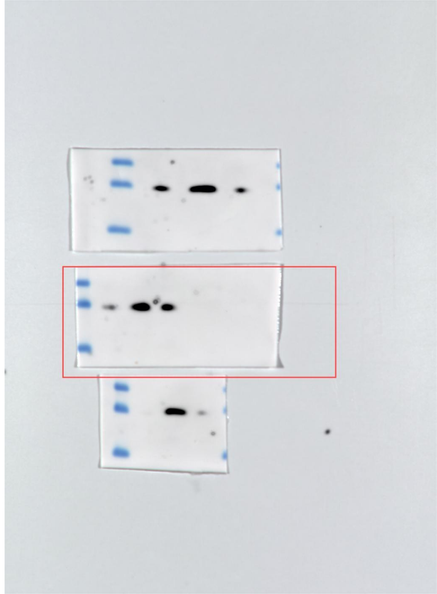

Figure 5D-30-p-CREB

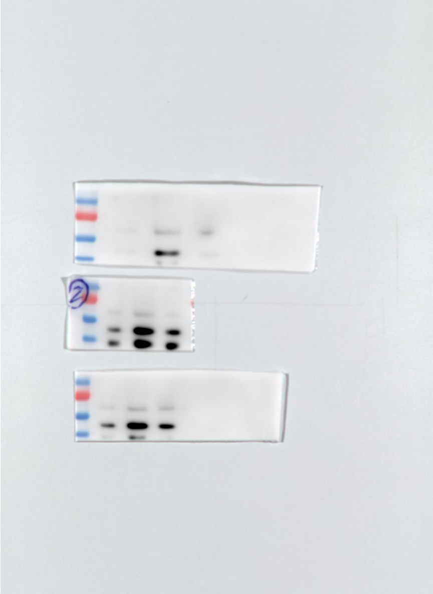

Figure 5D-30-ZIP8

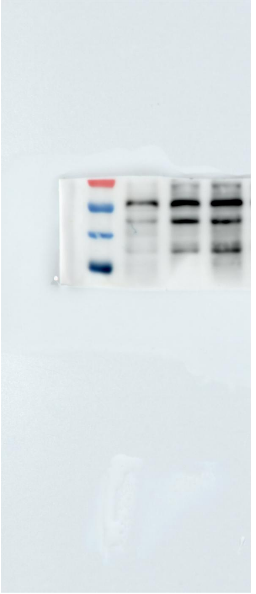

Figure 5D-450-actin

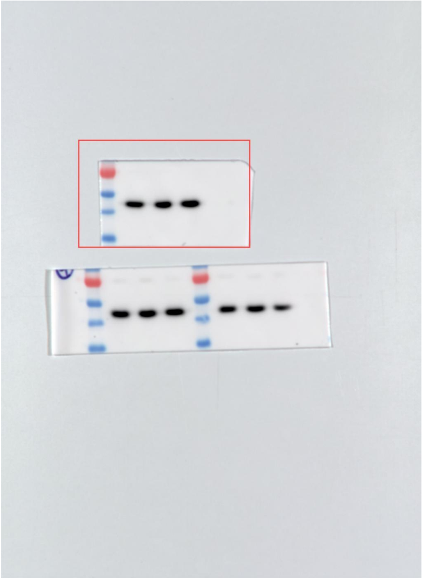

Figure 5D-450-CREB

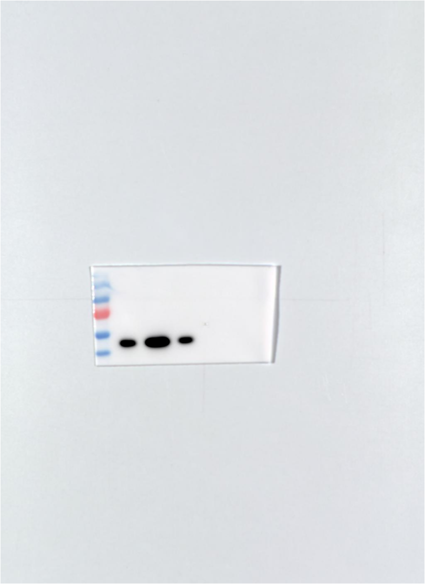

Figure 5D-450-GPX4

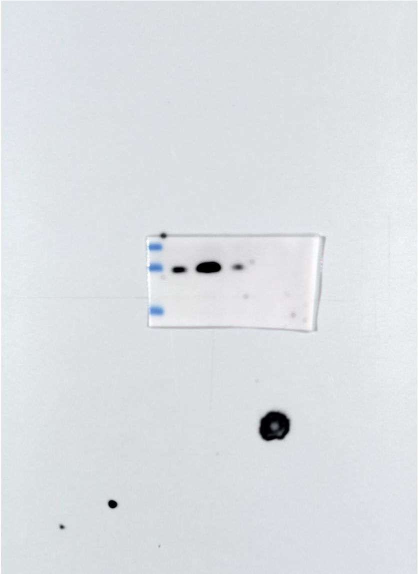

Figure 5D-450-p-CREB

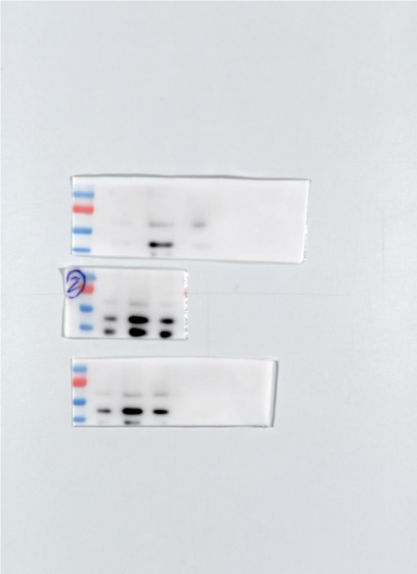

Figure 5D-450-ZIP8

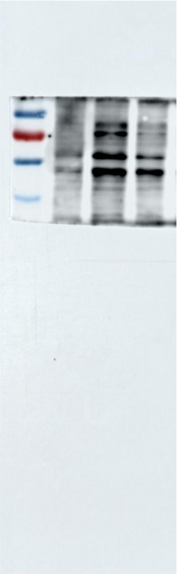

Figure 6D

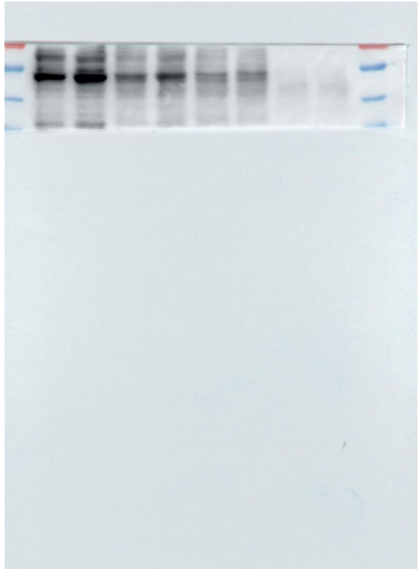

Figure 6E-down

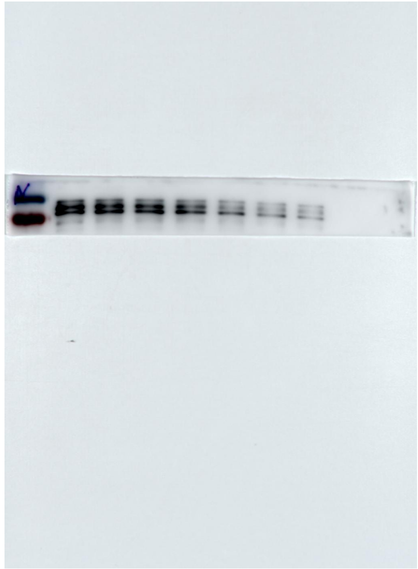

Figure 6E-up

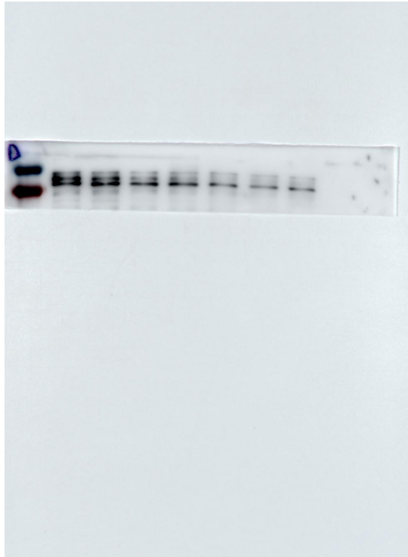

Figure 6F-down

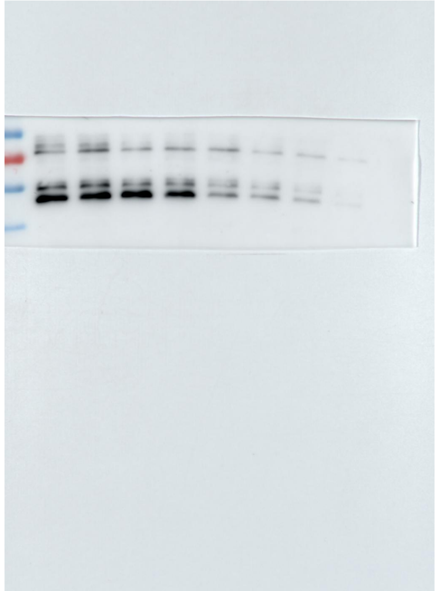

Figure 6F-up

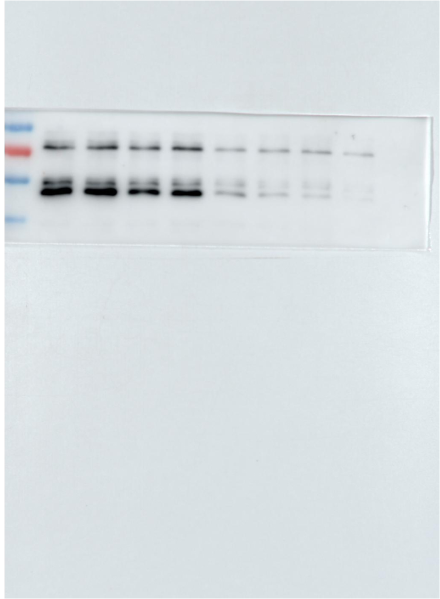

Figure 7C-30-actin

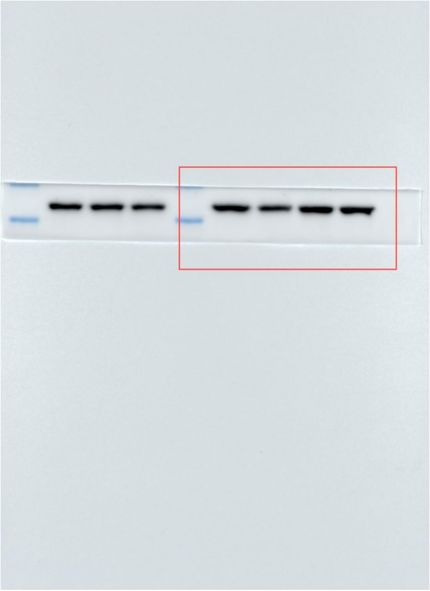

Figure 7C-30-FTH1

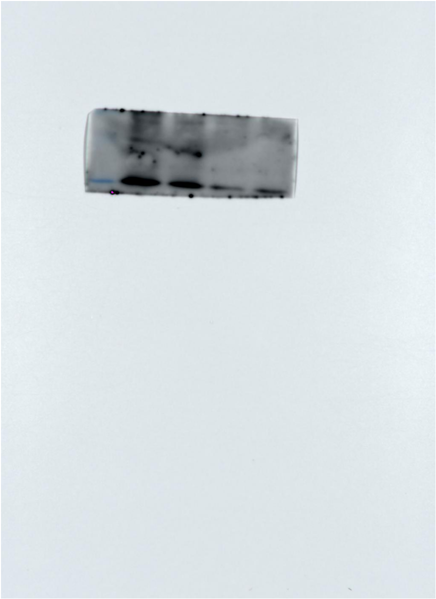

Figure 7C-30-FTL

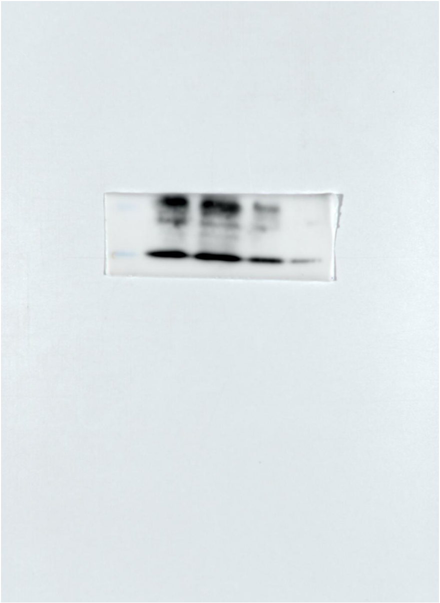

Figure 7C-30-ZIP8

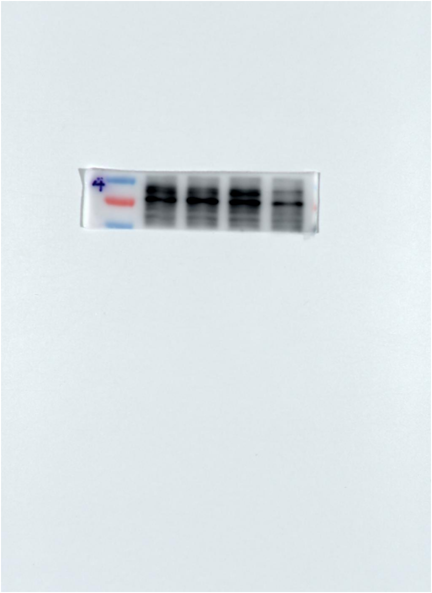

Figure 7C-450-actin

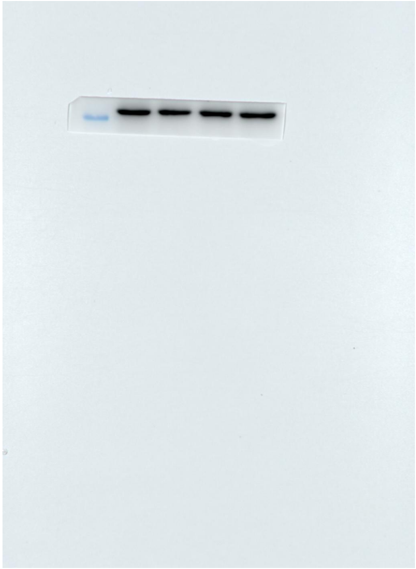

Figure 7C-450-FTH1

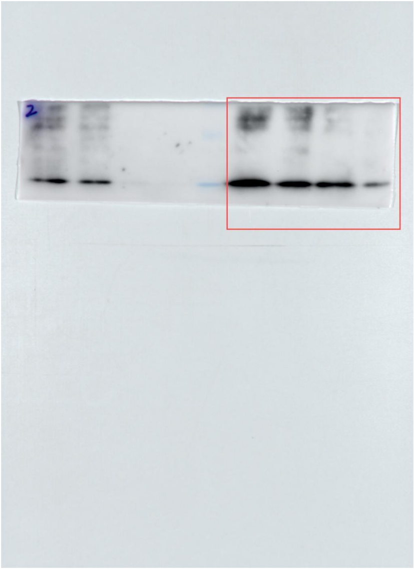

Figure 7C-450-FTL

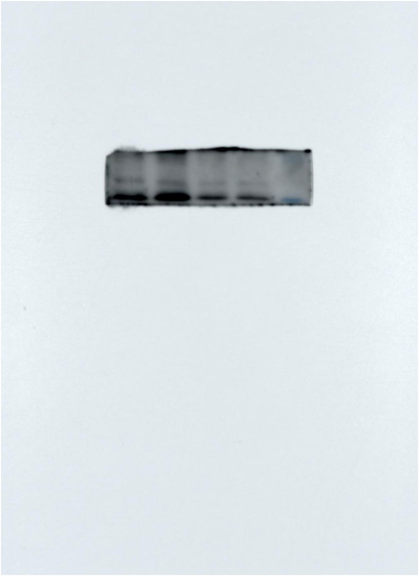

Figure 7C-450-ZIP8

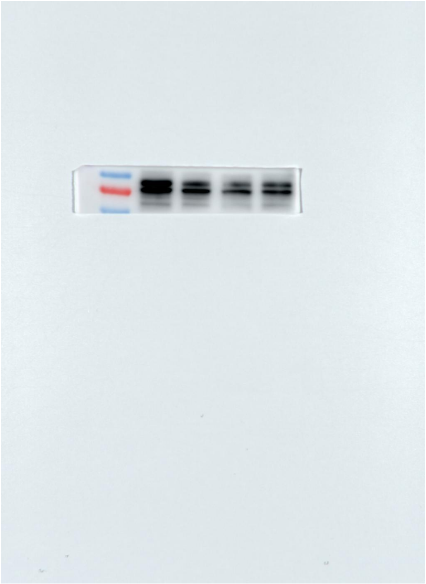

Figure 7C-510-FTH1

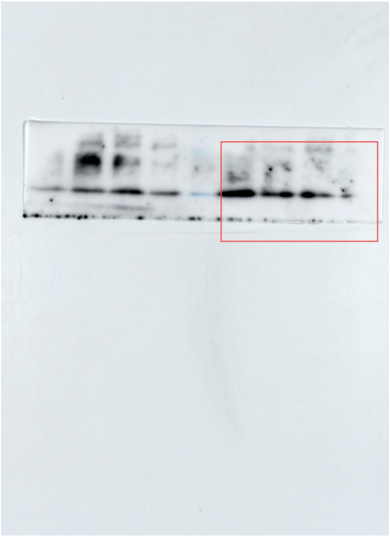

Figure 7C-510 FTL

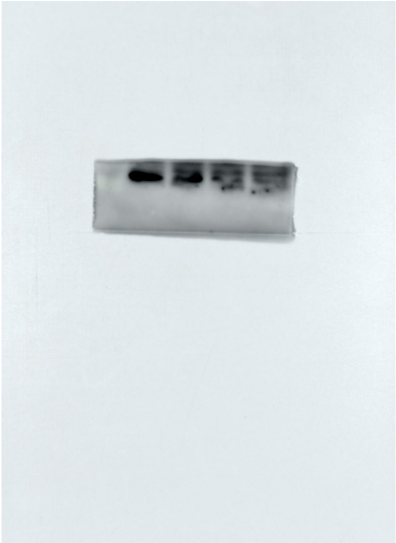

Figure 7C-510-actin

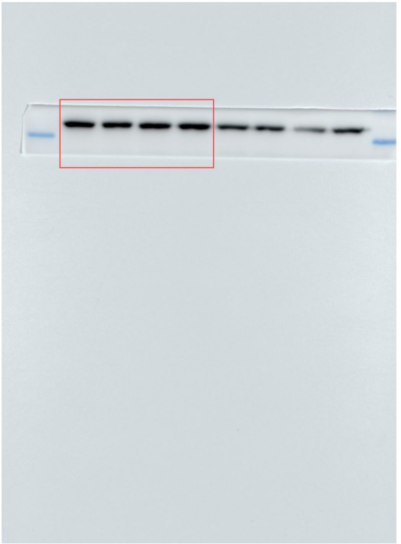

Figure 7C-510-ZIP8

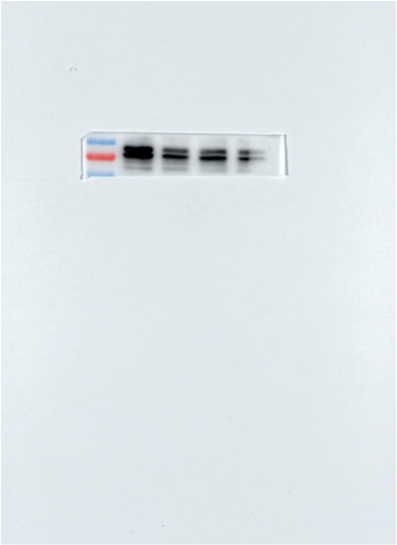

Figure 7F-30 - GPX4

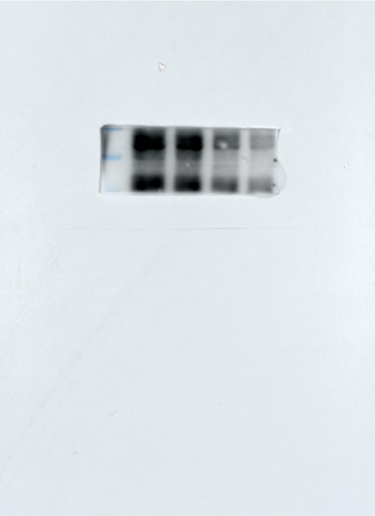

Figure 7F-30-actin

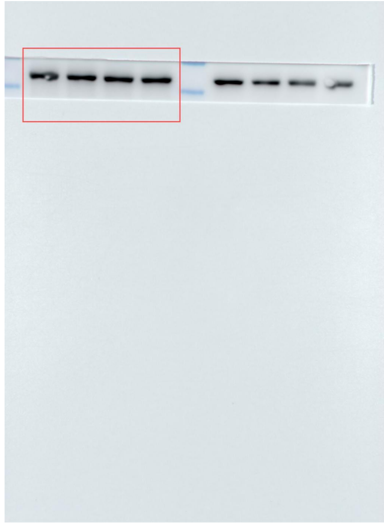

Figure 7F-30-CREB

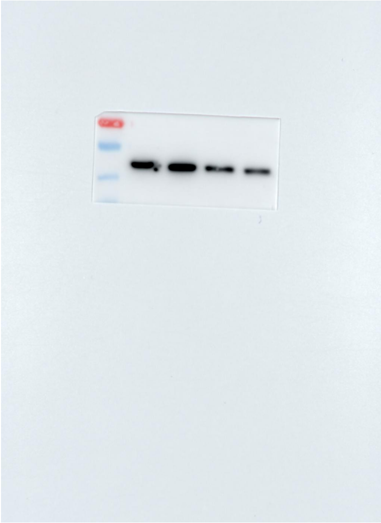

Figure 7F-30-p-CREB

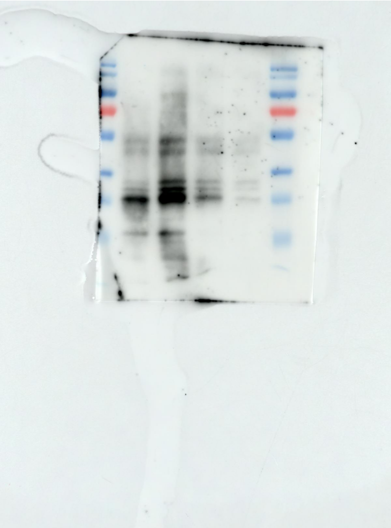

Figure 7F-30-ZIP8

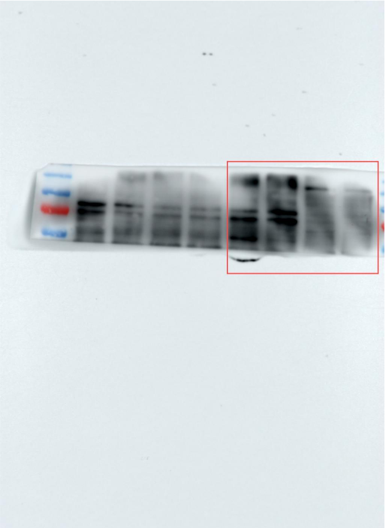

Figure 7F-450-actin

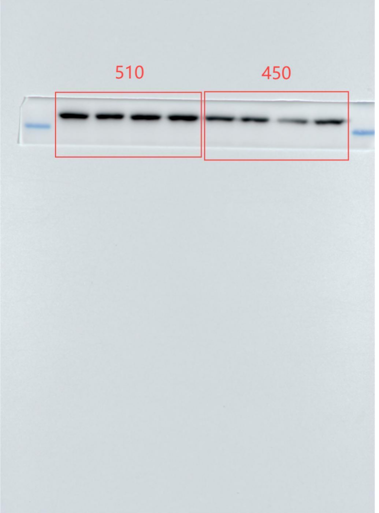

Figure 7F-450-CREB

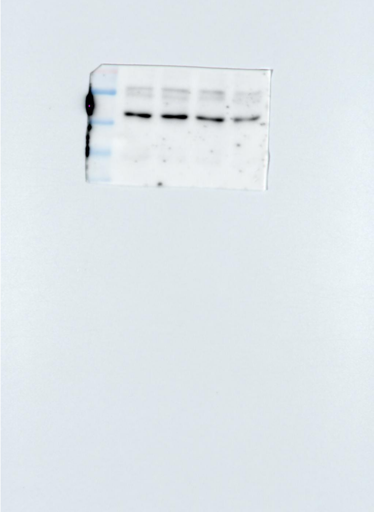

Figure 7F-450-GPX4

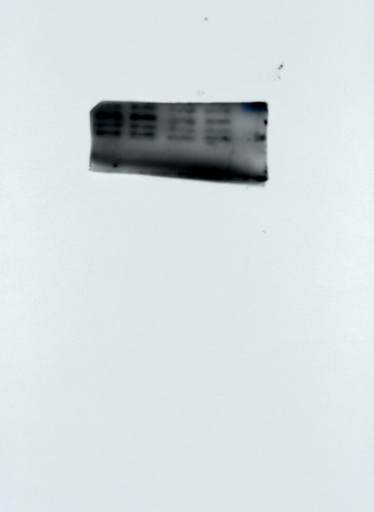

Figure 7F-450-p-CREB

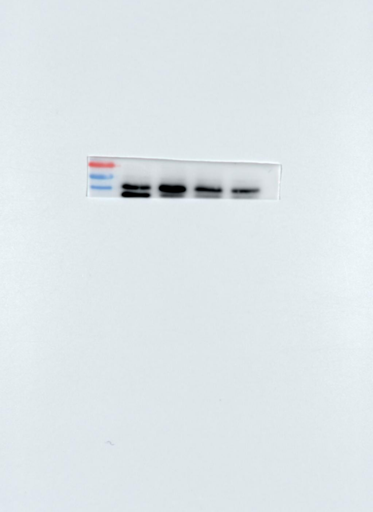

Figure 7F-450-ZIP8

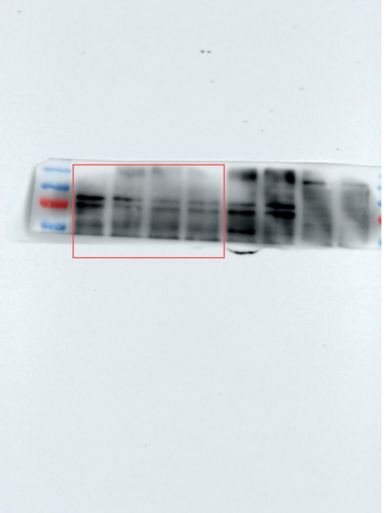

Figure 7F-510-actin

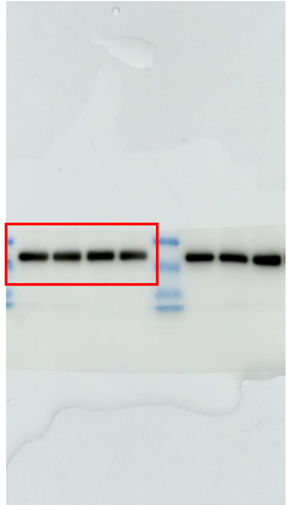

Figure 7F-510-CREB

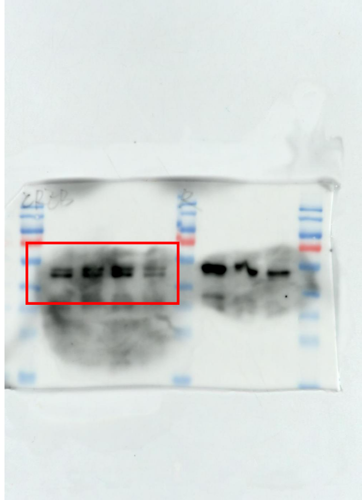

Figure 7F-510-GPX4

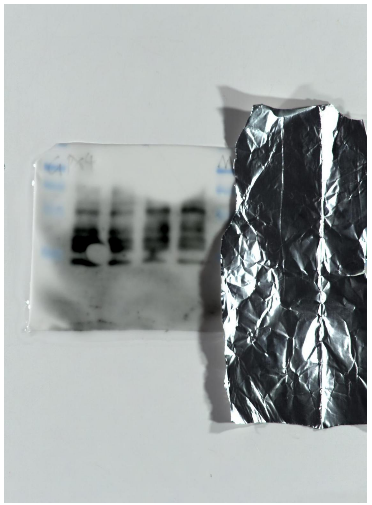

Figure 7F-510-p-CREB

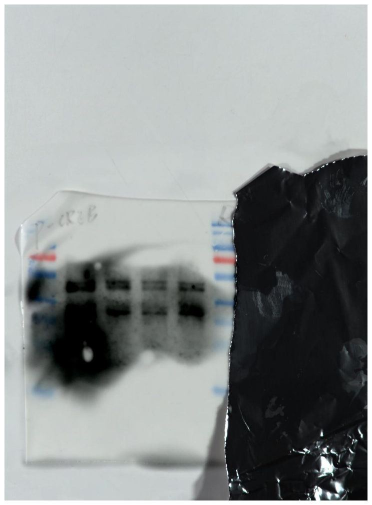

Figure 7F-510-ZIP8

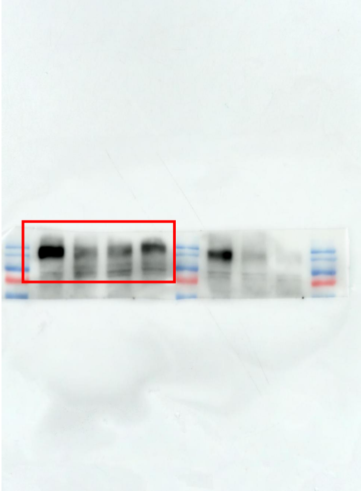

Figure S1-450 S133D

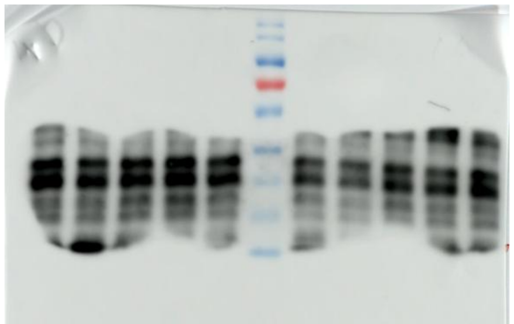

Figure S1-GAPDH

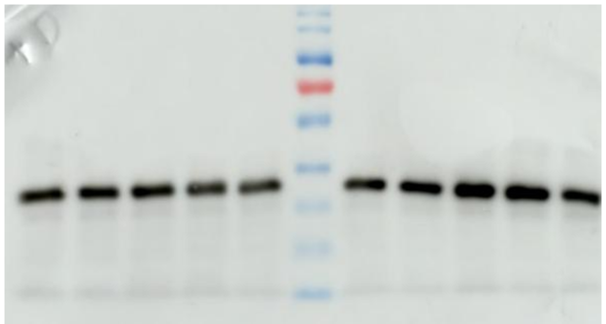

Figure S1-450 S133A

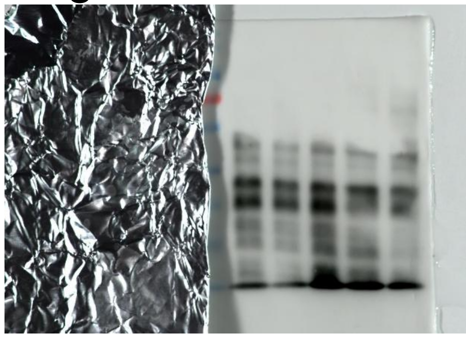

Figure S1-GAPDH

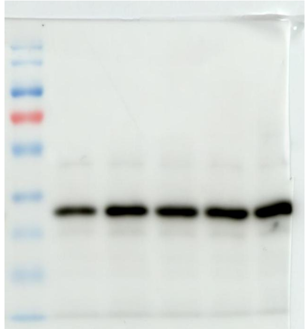

Figure S1-510 S133A

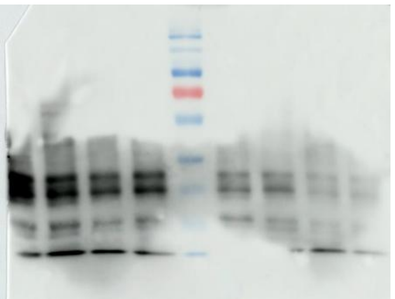

Figure S1- GAPDH

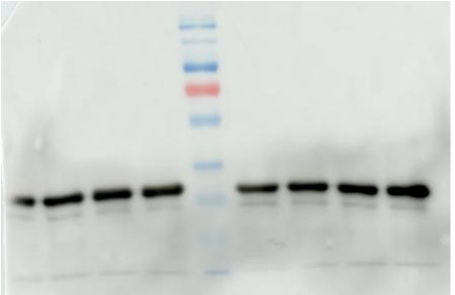

Figure S1-510 S133D

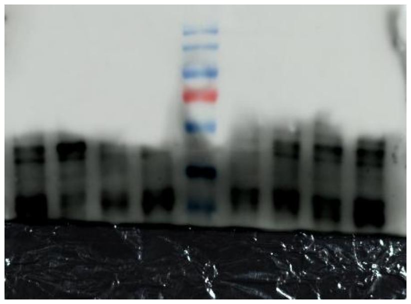

Figure S1-GAPDH

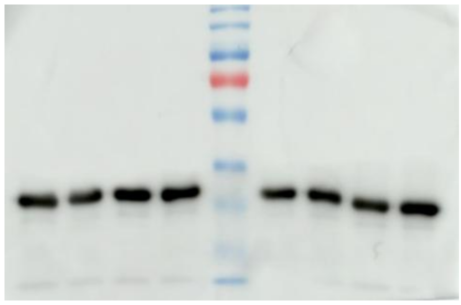

Figure S1-510 WT

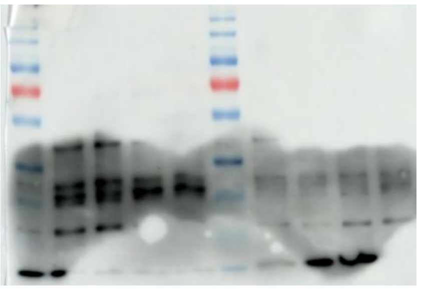

Figure S1-GAPDH

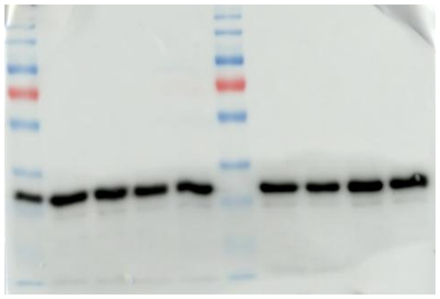

Supplement: Supplementary file 4 — original western blot images [file 41419_2025_7692_MOESM4_ESM.pdf]
